# Water/Fat Separated Echo Planar Time‐Resolved Imaging (WFS‐EPTI) for Distortion‐Free Multi‐Contrast MRI

**DOI:** 10.1002/mrm.70355

**Published:** 2026-04-09

**Authors:** Zhangxuan Hu, Timothy G. Reese, Lawrence L. Wald, Jonathan R. Polimeni, Zijing Dong, Fuyixue Wang

**Affiliations:** ^1^ Athinoula A. Martinos Center for Biomedical Imaging Massachusetts General Hospital Charlestown Massachusetts USA; ^2^ Department of Radiology Harvard Medical School Boston Massachusetts USA; ^3^ Harvard‐MIT Program in Health Sciences and Technology Massachusetts Institute of Technology Cambridge Massachusetts USA

**Keywords:** body imaging, chemical shift artifacts, echo‐planar imaging (EPI), fat saturation, fat suppression, subspace reconstruction

## Abstract

**Purpose:**

Echo‐planar time‐resolved imaging (EPTI) provides distortion‐ and T2/T2* blurring‐free multi‐echo/multi‐contrast imaging with fast speed, making it an efficient acquisition method for various MRI applications. Here, we aim to achieve water–fat separation for EPTI to improve fat signal removal for brain/body imaging applications.

**Theory and Methods:**

A water/fat separated EPTI (WFS‐EPTI) technique is developed, which leverages the intrinsic multi‐echo data provided by EPTI readout and introduces a novel strategy for highly‐accelerated spatiotemporal encoding and reconstruction to separate water and fat signals. Specifically, DIXON encoding is integrated into the spatiotemporal acquisition by acquiring the echo train at scheduled TEs and echo‐spacing, therefore modulating the rapidly changing fat phase evolution across EPTI readout into a more uniform, coherent manner (e.g., in‐phase and out‐of‐phase conditions for odd and even image series). This enables robust subspace representations even in the presence of fat signals. A joint subspace reconstruction is then proposed, which separately performs data consistency for each echo group to ensure fidelity, while jointly leveraging shared information to improve conditioning. Furthermore, an auto‐calibrated WFS‐EPTI is developed to improve motion robustness for abdominal imaging, and a data‐driven basis extraction is employed to address imperfect in‐phase/out‐of‐phase conditions.

**Results:**

Both phantom experiments and in vivo human imaging across multiple body regions—including brain, head–neck, and abdomen—demonstrated the feasibility of the proposed method.

**Conclusion:**

The proposed WFS‐EPTI can provide water/fat separation in rapid acquisition and obtain high resolution, distortion‐free multi‐contrast/quantitative imaging in the presence of fat signals.

## 1 Introduction

Echo‐planar imaging (EPI) [1] is a commonly used MR sequence in many applications because of its fast acquisition speed. However, it suffers from limitations such as T2* blurring, geometric distortions, and ghosting artifacts caused by gradient delays and anisotropic gradient delays. In addition, it is prone to chemical shift artifacts when fat saturation is inadequate, especially in body imaging [2, 3, 4, 5, 6, 7].

Commonly used fat saturation techniques include spectral‐selective methods, such as chemical shift selective (CHESS) [8], spectral pre‐saturation by inversion recovery (SPIR) [9], and spectral attenuated inversion recovery (SPAIR) [10], as well as other methods such as short TI inversion recovery (STIR) [11]. These methods generally provide effective fat saturation, but not in all situations. For example, spectral‐based methods are sensitive to B0 inhomogeneities [12, 13, 14], which can lead to insufficient fat suppression in challenging regions such as the neck–shoulder and spine areas [6, 15, 16]. Consequently, in EPI, where chemical shift artifacts are problematic—especially in body imaging, effective fat suppression remains a critical technical challenge for achieving high‐fidelity imaging.

As an alternative to fat saturation techniques, water/fat separation approaches have become increasingly popular. Water/fat separation based on chemical shift encoding utilizes the frequency differences between water and fat signals and models their phase evolution over time to separate their respective contributions. For example, the two‐point Dixon method [17] acquires two sets of images at slightly different echo times (TE): the first with fat and water signals in‐phase and the second with the TE adjusted by a few milliseconds so that the fat and water signals are out‐of‐phase. Water‐only and fat‐only images can then be computed from this pair of images. Numerous advancements based on the Dixon technique have since emerged, including the three‐point method [18] and the more advanced iterative decomposition of water and fat with echo asymmetry and least‐squares estimation (IDEAL), along with its subsequent modifications [19, 20, 21, 22, 23]. These advanced methods enable simultaneous estimation of B0 inhomogeneity alongside water and fat signals to accurately model and estimate the signal/phase evolutions.

However, for EPI based imaging, water/fat separation can be further complicated by chemical shift artifact: large fat‐signal shifts along the phase‐encoding direction cause fat signals superimposed on water images to originate from different locations and therefore experience different B0 inhomogeneities, making accurate modeling and separation more challenging. Combination of water/fat separation with single‐shot‐EPI (ss‐EPI) with more sophisticated modeling and correction strategies has demonstrated promising results [2, 3]. However, additional challenges remain, such as ss‐EPI's inherent limitations, T2* blurring and geometric distortions. While combining these water‐fat separation techniques with multi‐shot EPI (ms‐EPI) [24, 25, 26, 27, 28, 29, 30, 31] can mitigate these issues, it cannot fully eliminate them. Additionally, water/fat separation requires multiple repeated acquisitions with echo shifts, significantly increasing acquisition time, particularly in ms‐EPI.

Echo‐planar time‐resolved imaging (EPTI) [32, 33] addresses EPI's distortion and blurring by efficiently encoding and resolving signals across the readout through highly accelerated spatiotemporal acquisition and reconstruction. It retains the high sampling efficiency of EPI, while enabling distortion‐ and blurring‐free multi‐echo/multi‐contrast imaging across the readout with millisecond‐scale TE intervals. This makes EPTI an ultra‐fast and versatile tool for various brain MRI applications, including multi‐parametric quantitative imaging [33, 34, 35, 36], diffusion MRI [37, 38, 39], and functional MRI [40, 41, 42]. However, the challenge of effective fat saturation/separation needs to be addressed to enable its broader application in body imaging. Previous studies using EPTI have primarily relied on fat‐saturation techniques (e.g., CHESS). Methods for water–fat separation that leverage its intrinsic multi‐echo information remain largely unexplored, which can potentially further improve the removal of fat‐related signals and artifacts.

In this study, we propose a water/fat separated EPTI (WFS‐EPTI) technique, which leverages the intrinsic multi‐echo data provided by EPTI readout and proposes a novel strategy in highly accelerated spatiotemporal encoding and reconstruction to separate water and fat signals. This method allows water/fat separation in rapid acquisition and provides high resolution, distortion‐free multi‐contrast/quantitative imaging. We demonstrate the feasibility of this novel approach through both phantom experiments and in vivo human imaging across multiple body regions, including the brain, head/neck, and abdomen, all in the absence of fat saturation modules, as a proof of concept to demonstrate the efficacy of achieving high‐fidelity EPTI images in the presence of fat signals.

## 2 Theory

### 2.1 EPTI Acquisition and Subspace Reconstruction

**FIGURE 1:**
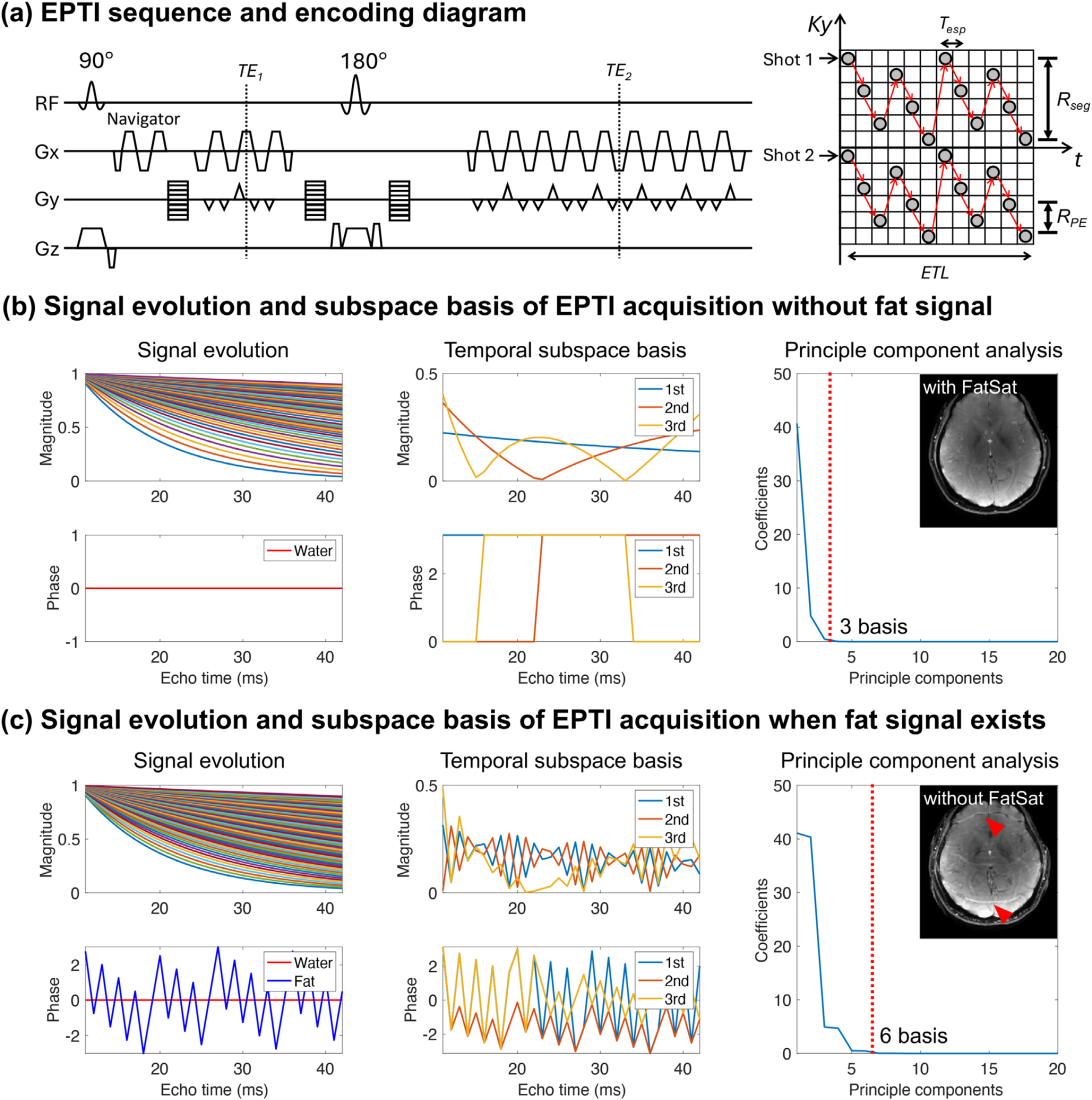
Overview of conventional EPTI acquisition and subspace reconstruction. (a) Combined gradient‐echo and spin‐echo (GESE) EPTI sequence diagram: After a standard three‐line one‐dimensional navigator, EPTI readout is acquired, where gradient blips along the phase‐encoding direction (*G*_*y*_) are used to achieve spatiotemporal CAIPI sampling trajectories. The corresponding EPTI encoding pattern in *k*_*y*_ *‐t* space is shown on the right: Echo‐train length of the readout is denoted by ETL; readout lines are spaced apart in time by the echo spacing (*T*_esp_) and in the phase‐encoding (PE) direction by *R*_*PE*_; and *R*_*seg*_ denotes the coverage along PE direction of each EPTI shot. Simulated signal evolutions and subspace analysis performed for a gradient‐echo EPTI (b) without fat signals (e.g., when fat signals are sufficiently suppressed by FatSat modules) and (c) with fat signals (e.g., without applying FatSat or insufficient fat suppression). The left column presents simulated magnitude and phase evolutions of water (red) and fat (blue) signals across TE. The middle column illustrates the first three temporal basis components derived from singular value decomposition (SVD). The right column shows the corresponding singular value spectra (coefficients), with the number of significant basis components indicated by the red dashed lines. Insets show representative reconstructed brain images from data acquired with and without applying fat saturation using conventional subspace reconstruction, highlighting noticeable fat artifacts (red arrows) in the absence of fat saturation.

Figure 1a shows the sequence diagram of a gradient‐echo and spin‐echo (GESE) EPTI and the corresponding encoding pattern in *k*_*y*_ *‐t* space. In EPTI, the readout lines are spaced apart in time by an echo spacing (*T*_esp_) and in the phase‐encoding (PE) direction by *R*_PE_, with *R*_PE_ = 1 corresponding to the fully sampled case. *R*_seg_ denotes the coverage along the PE direction of each EPTI shot. Spatiotemporal controlled aliasing in parallel imaging (CAIPI) sampling is employed by alternating the PE encoding for different readout lines in a spatiotemporal complementary pattern. With EPTI, images at all echo times along the readout echo train—each separated by the echo spacing (ESP)—are reconstructed. Since all phase‐encoding lines for each image are at the same echo time, there is no distortion along the phase‐encoding direction in EPTI acquisitions.

To improve the ability to accelerate while maintaining image reconstruction performance, a subspace reconstruction [33, 43, 44] algorithm was developed for EPTI [33]. As shown in Figure 1b, signal evolution space within specific quantitative parameter (T2* in this case) value ranges is firstly simulated based on the acquisition parameters. Then, a small set of basis vectors (e.g., three basis vectors in Figure 1b middle) denoted as ϕ is extracted through principal component analysis (PCA). These vectors form a low‐dimensional subspace (Figure 1b right) that can closely approximate the entire signal space of interest. Using these basis vectors, the image series across the readout echo train can be calculated by ϕc, where *c* is the coefficient maps of the basis vectors. In this way, the number of unknowns in the reconstruction are reduced from the number of echoes to the number of basis vectors, improving the reconstruction conditioning and thus the image SNR. The subspace reconstruction is computed by

(1) $$\min_{c}{\left\|\text{UFSBϕ}c-y\right\|}_{2}^{2}+\lambda R\left(c\right)$$

where ***B*** is the phase evolution across different image echoes due to B_0_ inhomogeneity; ***S*** is the coil sensitivity; ***F*** is the Fourier transform operator; ***U*** is the undersampling mask; and *y* is the acquired undersampled *k*‐*t* space dataset. A fully sampled (*k*_*x*_ *‐k*_*y*_ *‐t*) low‐resolution calibration data is acquired to estimate the sensitivity maps and B0 maps, which are used to obtain ***B*** and ***S***. The regularization term ***R*** (*c*) can be incorporated to further improve the conditioning, and λ is the control parameter of the regularization. A locally low‐rank (LLR) regularization in image domain [45] or Hankle matrix based regularization in *k*‐space domain [46] can be used. After solving for *c*, images across echo train can be produced by the product of ϕc.

### 2.2 Fat Signals in Subspace Reconstruction of EPTI

Subspace reconstruction can improve the efficiency of EPTI; however, low‐rank behavior of the signal evolution is essential for robust reconstruction performances under high accelerations, and this low‐rank structure can be disrupted when fat signals are present—for example, in the absence of fat saturation or when fat suppression is insufficient. As a simplification, we consider a single‐peak fat spectrum (typically the main fat peak at 3.40 ppm). Taking gradient‐echo signal as an example, the image $I_{m}$ at the (*m* + 1)‐*th* echo can be expressed as:

(2) $$I_{m}=\left(\rho_{w}+\rho_{F}e^{-i2\pi f_{F}\left(t_{0}+mT_{\mathrm{esp}}\right)}\right)e^{-\left(t_{0}+mT_{\mathrm{esp}}\right)/T_{2}^{*}}e^{-i\gamma ∆B\left(t_{0}+mT_{\mathrm{esp}}\right)}$$

where $\rho_{w}$ is the complex‐valued intensity of the water component; $\rho_{F}$ is the complex‐valued intensity of the fat component with a frequency shift $f_{F}$ (in Hz); $t_{0}$ is the echo time of the first echo; $T_{\mathrm{esp}}$ is the ESP; $e^{-i2\pi f_{F}\left(t_{0}+mT_{\mathrm{esp}}\right)}$ describes the chemical‐shift induced phase evolution of the fat signals; $e^{-\left(t_{0}+mT_{\mathrm{esp}}\right)/T_{2}^{*}}$ represents the $T_{2}^{*}$ decay; $e^{-i\gamma ∆B\left(t_{0}+mT_{\mathrm{esp}}\right)}$ accounts for phase caused by B0 inhomogeneity.

In this case, the subspace bases are required to represent the signal evolution of both water and fat. Figure 1c shows the simulated signal evolutions and the corresponding subspace bases. The rapidly evolving fat phase $e^{-i2\pi f_{F}\left(t_{0}+mT_{\mathrm{esp}}\right)}$ compromises the low‐rank nature, requiring a larger and more complex set of basis vectors and leading to a more challenging reconstruction problem. Even with these more complex bases, fat‐related artifacts may persist and degrade the reconstructed images (as highlighted by the red arrows in Figure 1c).

### 2.3 WFS *‐* EPTI Acquisition and Reconstruction

**FIGURE 2:**
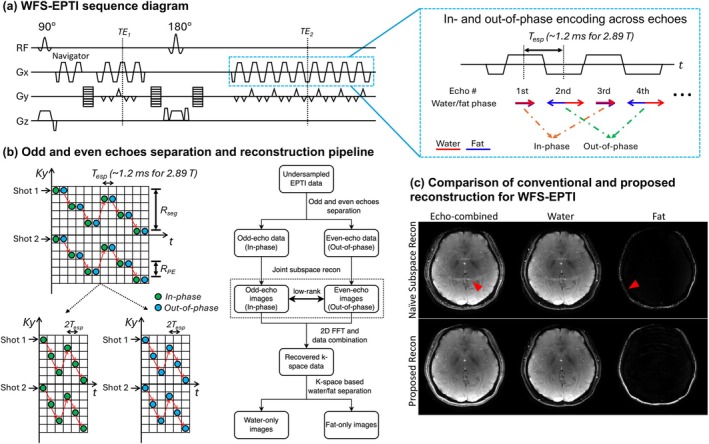
Schematic diagram illustrating the proposed water/fat separated EPTI (WFS‐EPTI) acquisition and reconstruction workflow. (a) Sequence diagram of the combined gradient‐echo and spin‐echo (GESE) WFS‐EPTI acquisition, highlighting the echo‐spacing scheduled spatiotemporal sampling strategy. The echo spacing (*T*_esp_) is chosen (∼1.2 ms at 2.89 Tesla) to modulate fat phase evolution across the readout into a more coherent manner, such that the odd and even echoes acquire in‐phase and out‐of‐phase image series respectively, each exhibiting consistent phase behavior that can be more effectively represented by subspace basis. (b) Sampling trajectory and reconstruction pipeline: In WFS‐EPTI, spatiotemporal CAIPI sampling trajectories are performed separately for odd and even echoes, thus consecutive pairs of odd and even echoes sample identical *k*‐space locations. The undersampled WFS‐EPTI data are first separated into odd‐echo (in‐phase) and even‐echo (out‐of‐phase) datasets. Images are then reconstructed using a joint reconstruction approach, leveraging the low‐rank properties of both odd and even echoes for improved conditioning while enforcing data consistency separately to account for fat signals. The reconstructed images are subsequently transformed into the *k*‐space domain, where a *k*‐space‐based water/fat separation technique is applied to obtain water‐only and fat‐only images. (c) Comparison between conventional and proposed reconstruction of a gradient‐echo WFS‐EPTI dataset acquired without fat saturation. The data were acquired with resolution = 1 × 1 × 3 mm^3^, acquisition matrix = 204 × 180, 10 slices, flip angle = 90°, TR = 2000 ms, TE = 10.4–54.8 ms, ETL = 38, 9 shots, acceleration factor (*R*_*seg*_ */R*_*PE*_) = 20/4. The proposed method effectively removes fat artifacts (red arrows) compared to conventional reconstruction.

To address the rapidly evolving fat phase in spatiotemporal acquisition and reconstruction, Dixon's two‐point encoding [17] is incorporated into the data acquisition and reconstruction of the proposed WFS‐EPTI technique. This involves modulating the baseline fat phase evolution across readout into a more coherent manner by acquiring the echo train at scheduled TEs and echo‐spacing. For example, choosing the echo‐spacing to be equal to, or a multiple of, half the period of the fat chemical shift period (e.g., *T*_*esp*_ = 1.2 ms at 2.89 Tesla, determined by the chemical shift of the primary fat spectral peak at 3.40 ppm), so that the fat signal evolution exhibits consistent phase behavior across odd‐echo or even‐echo image series respectively (i.e., acquire in‐phase and out‐of‐phase image series, respectively) (Figure 2a). Then, the image series acquired with WFS‐EPTI can be defined as:

(3) $$I_{m}=\left\{\begin{array}{l} \left(\rho_{w}+\rho_{F}\right)e^{-\left(t_{0}+mT_{\mathrm{esp}}\right)/T_{2}^{*}}e^{-i\gamma ∆B\left(t_{0}+mT_{\mathrm{esp}}\right)}\;m\in \mathrm{odd}\;\text{echoes}\\ \left(\rho_{w}-\rho_{F}\right)e^{-\left(t_{0}+mT_{\mathrm{esp}}\right)/T_{2}^{*}}e^{-i\gamma ∆B\left(t_{0}+mT_{\mathrm{esp}}\right)}\;m\in \text{even echoes} \end{array}\right.$$

Thus, the fat‐changing phase term $e^{-i2\pi f_{F}\left(t_{0}+mT_{\mathrm{esp}}\right)}$ and its associated effects on the signal evolution of the odd‐ and even‐echo image series are effectively eliminated. Consequently, the signal evolution in both image series is represented solely by $T_{2}^{*}$ decay, preserving the low‐rank property and allowing effective representation with a reduced number of subspace basis. Additionally, the encoding pattern in WFS‐EPTI is optimized by independently applying the spatiotemporal CAIPI trajectory for odd and even echoes, ensuring consistency between in‐phase and out‐of‐phase image series, that is, consecutive pairs of odd and even echoes sample identical *k*‐space locations (Figure 2b).

Figure 2b right shows the flowchart of the WFS‐EPTI reconstruction, where a joint regularized subspace reconstruction approach is developed. The in‐phase and out‐of‐phase data are solved separately to maintain their respective data consistency but regularized jointly to leverage shared structural/contrast information between them for improved reconstruction conditioning and SNR, as detailed below:

(4) $$\begin{array}{cc} & \min_{c_{\mathrm{odd}},c_{\text{even}}}{\left\|\text{UFSB}\phi_{\mathrm{odd}}c_{\mathrm{odd}}-y_{\mathrm{odd}}\right\|}_{2}^{2}+{\left\|\text{UFSB}\phi_{\text{even}}c_{\text{even}}-y_{\text{even}}\right\|}_{2}^{2}\\ & \;+\lambda R\left(c_{\mathrm{odd}},c_{\text{even}}\right) \end{array}$$

where $\phi_{\mathrm{odd}}$ and $\phi_{\text{even}}$ are subspace basis generated for the odd and even echoes, respectively; $c_{\mathrm{odd}}$ and $c_{\text{even}}$ are the corresponding coefficient maps; $y_{\mathrm{odd}}$ and $y_{\text{even}}$ are the acquired *k*‐*t* space datasets for odd and even echoes. While enforcing the data consistency separately for the odd and even echoes to account for the presence of fat signals, the regularization term $R\left(c_{\mathrm{odd}},c_{\text{even}}\right)$ is applied jointly for both sets of images. In this study, image‐domain LLR regularization [45] was applied to the combined coefficient maps of $c_{\mathrm{odd}}$ and $c_{\text{even}}$, in contrast to applying LLR separately to $c_{\mathrm{odd}}$ and $c_{\text{even}}$. Figure 2c shows an example of the proposed method compared with the naive subspace reconstruction (i.e., subspace reconstruction using bases generated with fat signals included) on a representative WFS‐EPTI dataset acquired without fat saturation. Fat‐related artifacts observed in the conventional reconstruction (indicated by the red arrows) were effectively eliminated with the proposed approach, resulting in clean and well‐separated water‐only and fat‐only images.

After subspace reconstruction, water/fat separation algorithms [23, 47, 48] can be applied to the reconstructed multi‐echo images to generate water‐only and fat‐only images, as well as B0 maps. In this study, the reconstructed images are transformed into *k*‐space and a *k*‐space based water/fat separation algorithm [49, 50] is employed, in which a multipeak fat spectral model is considered [51, 52]. This algorithm also corrects for potential chemical‐shift misregistration of water and fat signals along the *readout* direction—caused by the bipolar readout in echo‐planar acquisition—by accounting for the acquisition timing differences between odd and even echo readout samples.

### 2.4 Auto‐Calibrated WFS‐EPTI Acquisition With Data‐Driven Basis Extraction

#### 2.4.1 Auto‐Calibrated Acquisition

An auto‐calibrated WFS‐EPTI acquisition is developed for imaging regions affected by physiological motion (e.g., respiratory motion), with the goals to integrate the imaging and calibration scans so that they share the same contrast and motion state (e.g., breath‐hold positions).

**FIGURE 3:**
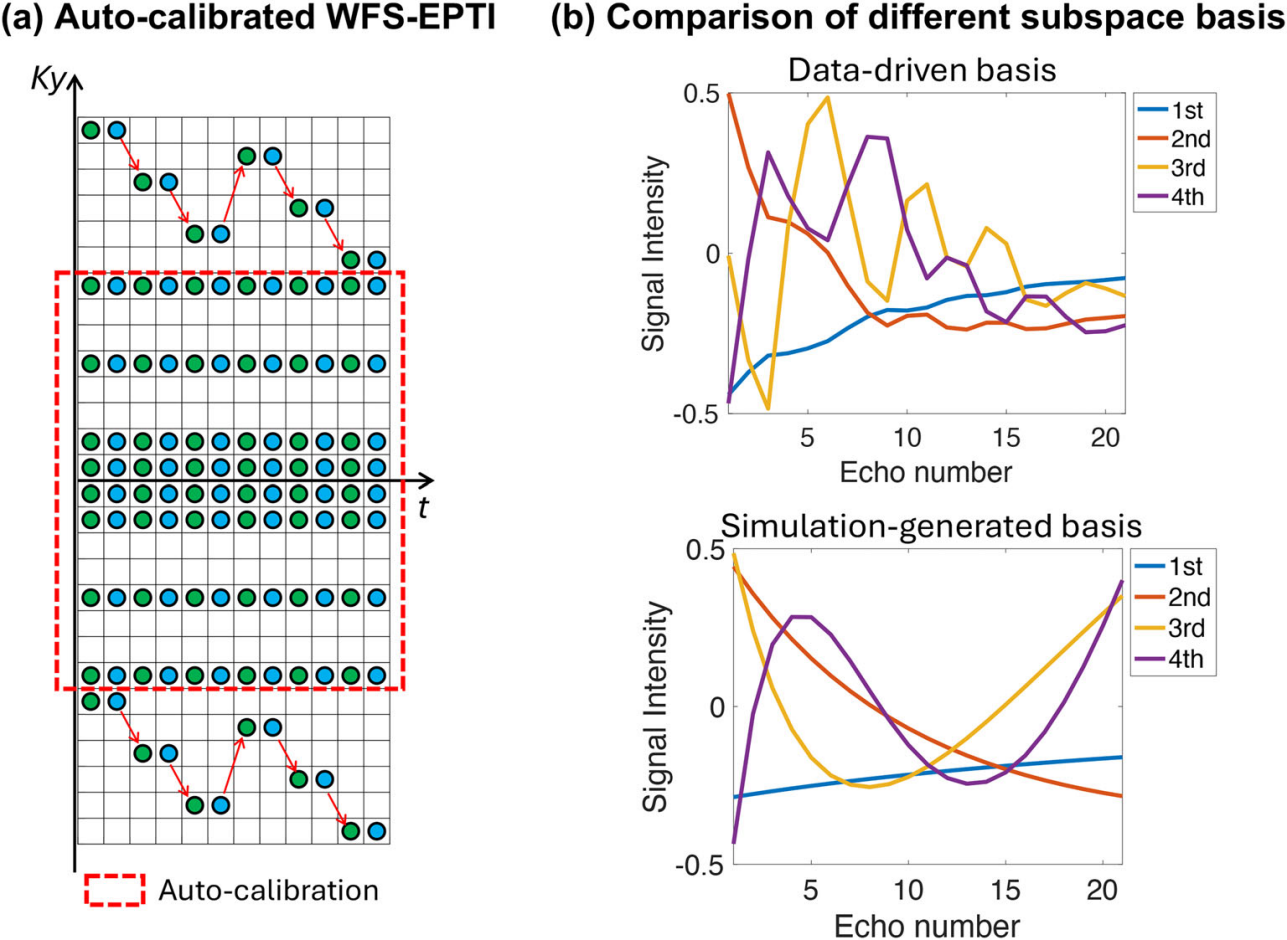
Auto‐calibrated WFS‐EPTI acquisition. (a) In the auto‐calibrated WFS‐EPTI, the central of the *k*_*y*_ *‐t* space region (indicated by the red dashed rectangle) is densely sampled, with fully sampled center lines and moderately accelerated adjacent lines. The remaining peripheral regions are highly accelerated using the spatiotemporal CAIPI trajectory, as in conventional EPTI acquisitions. (b) Data‐driven bases generated from the auto‐calibration data (top) are able to capture signal fluctuations caused by imperfect in‐phase/out‐of‐phase conditions specific to the dataset, compared to simulation‐generated bases that assume perfect phase conditions (bottom).

Similarly to parallel imaging, EPTI reconstruction requires a calibration scan to estimate coil sensitivity and B0 inhomogeneity, typically acquired using a short low‐resolution, fully sampled gradient‐echo pre‐scan. Here, the auto‐calibrated WFS‐EPTI embeds calibration within the imaging scan by employing a non‐uniform undersampling pattern that oversamples the *k*‐space center (Figure 3a). Specifically, the central region of *k*_*y*_ *‐t* space is densely sampled, with fully sampled center lines and moderately accelerated adjacent regions, while the remaining periphery is highly accelerated using the spatiotemporal EPTI trajectory. During reconstruction, the central auto‐calibration region is first recovered using GRAPPA [53] at each echo time. These auto‐calibration data, acquired under the same contrast and motion state as the imaging data, are then used for coil and B0 calibration and for data‐driven subspace basis extraction described below.

Since embedding calibration data within the auto‐calibrated WFS‐EPTI increases the acquisition time per breath‐hold, we also evaluate externally calibrated WFS‐EPTI with data‐driven basis extraction, where calibration data from one repetition—acquired during a different breath‐hold—is used for the calibration and subspace basis extraction of another repetition.

#### 2.4.2 Data‐Driven Basis Extraction to Address Imperfect In‐Phase/Out‐Of‐Phase Conditions

A data‐driven subspace basis extraction is developed to capture signal evolution even when imperfect in‐phase/out‐of‐phase condition occurs due to non‐ideal echo‐spacing and/or other chemical‐shift frequencies.

Imperfect in‐phase/out‐of‐phase conditions lead to additional signal/phase evolution across echoes from fat signal, therefore, more bases might be required to represent the signal evolution, and they become more complex (e.g., multi‐peak chemical‐shift frequencies of fat and their impact on the subspace bases are illustrated in Figure S1). The data‐driven basis is therefore designed to capture these complex components while reducing the number of required bases by tailoring the basis generation to the characteristics of the acquired dataset (rather than relying on bases generated from predefined multi‐peak fat spectrum simulation). Specifically, the data‐driven bases are extracted from the auto‐calibration data rather than generated from a simulated dictionary, and they are designed to be complex‐valued. Separate bases were computed for the odd and even echoes. As shown in Figure 3b top panel, they captured signal fluctuations resulting from imperfect in‐phase/out‐of‐phase conditions, in contrast to the simulation‐generated bases that assume perfect in‐phase/out‐of‐phase conditions shown in the bottom panel. Consequently, using these bases improves the performance of subspace reconstruction, as demonstrated in Figure S2.

## 3 Methods

Experiments were conducted using a whole‐body 3T (actual field strength is 2.89T) scanner (Prisma, Siemens Healthineers, Erlangen, Germany). All volunteers provided written informed consent prior to the scanning, in accordance with all the policies of our institution's Human Subjects Research Committee. Both phantom and in vivo experiments were performed to evaluate the proposed method. For WFS‐EPTI acquisition, the *T*_*esp*_ (≈1.2 ms) was determined based on the chemical shift of fat signals at 2.89 Tesla. In gradient‐echo WFS‐EPTI, the TEs were selected as close as possible to those satisfying the in‐phase/out‐of‐phase conditions, within the constraints of the echo train length (ETL). All *k*‐space data were reconstructed offline in MATLAB (Mathworks Inc., Natick, MA), following the workflow shown in Figure 2b. All *k*‐space data underwent odd‐even echo phase correction to remove the readout line misalignment effect before subspace reconstruction. The optimization problem was solved using the alternating direction method of multipliers (ADMM) algorithm [54]. The simulated subspace bases were generated for odd and even echoes separately from signal evolutions with T2/T2* values ranging from 10 to 500 ms for brain and head–neck imaging, and from 5 to 300 ms for body imaging applications. By combining Dixon encoding to remove phase modulation from the chemical shift (for the main fat peak), we simulated only T2/T2* relaxation effects for basis calculation. For the simulated subspace, three basis vectors were extracted, whereas for the data‐driven subspace, four basis vectors were derived from the acquired low‐resolution auto‐calibration *k*‐space data.

### 3.1 Phantom Experiments

To evaluate the feasibility of the proposed WFS‐EPTI method, a custom‐built water/fat phantom was scanned using a 32‐channel head‐coil. The phantom consisted of three tubes filled with fat and ten tubes filled with water. Gradient‐echo WFS‐EPTI data were acquired with parameters: resolution = 1 × 1 × 3 mm^3^, acquisition matrix = 204 × 204, 10 slices, TR = 800 ms, TE range = 8–56 ms, ETL = 41, *R*_*seg*_/*R*_*PE*_ = 12/4, number of shots = 17, no fat saturation, scan time = 14 s. The water/fat images and B0 maps estimated from WFS‐EPTI were further compared with those obtained using a conventional multi‐echo GRE sequence. The multi‐echo GRE was acquired with the same spatial coverage and resolution as WFS‐EPTI, and six evenly spaced echoes (TE ranging from 2.46 to 20.91 ms) were acquired, with a total scan time of 54 s. A Graph‐cut algorithm [51] was used for water/fat separation of images from the multi‐echo GRE scan.

### 3.2 In Vivo Experiments

Six healthy volunteers (five males; age 28 ± 4 years) were scanned. Two underwent brain and head–neck imaging, and four underwent abdominal imaging.

#### 3.2.1 Experiment 1: Retrospective Undersampling Experiment

Fully sampled gradient‐echo WFS‐EPTI data were acquired in the head–neck region and used to evaluate the performance of different reconstruction approaches under different acceleration factors. The acquisition parameters were: resolution = 1 × 1 × 3 mm^3^, FOV = 256 × 206 mm^2^, 10 slices, flip angle = 90°, TR = 800 ms, TE range = 13–62.2 ms, ETL = 42, number of shots = 206 (fully‐sampled *k*_*y*_ *‐t* space), no fat saturation, scan time = 2 min 45 s. The fully‐sampled data were then retrospectively undersampled with six different acceleration factors, corresponding to six acquisitions using different numbers of shots = 5, 7, 9, 11, 13, and 15, which yielded scan times of 4.0, 5.6, 7.2, 8.8, 10.4, and 12.0 s, respectively. Three reconstruction approaches were compared: (1) the conventional naive EPTI reconstruction, which uses subspace reconstruction with a basis set generated from signals that include fat; (2) the separated WFS‐EPTI reconstruction, where the odd and even echo images were reconstructed independently using subspace reconstruction and regularized separately; (3) the joint WFS‐EPTI reconstruction, where the odd and even echo images were jointly regularized. The fully sampled images were used as the references to calculate error maps and corresponding normalized root mean squared errors (nRMSEs).

#### 3.2.2 Experiment 2: Head and Neck Imaging With WFS‐EPTI

Brain images were acquired using 9‐shot and 17‐shot simultaneous GESE WFS‐EPTI sequences with a 32‐channel head coil. The acquisition parameters were as follows: resolution = 1 × 1 × 3 mm^3^, acquisition matrix = 204 × 204, 10 slices, flip angle = 90°, TR = 2000 ms, TE range for gradient echoes = 10.4–54.8 ms, TE range for spin echoes = 86.4–166.8 ms, ETL1/ETL2 = 38/68, no fat saturation. *R*_*seg*_/*R*_*PE*_ was 20/4 for the 9‐shot acquisition (scan time = 18 s) and 12/4 for the 17‐shot acquisition (scan time = 34 s).

Head–neck images were acquired using 9‐shot and 15‐shot simultaneous GESE WFS‐EPTI sequences with a 64‐channel head–neck coil. The acquisition parameters were as follows: resolution = 1 × 1 × 3 mm^3^, acquisition matrix = 256 × 180, 10 slices, flip angle = 90°, TR = 2000 ms, TE range for gradient echoes = 12.8–52.4 ms, TE range for spin echoes = 77.6–155.6 ms, ETL1/ETL2 = 34/66, no fat saturation. *R*_*seg*_/*R*_*PE*_ was 20/4 for the 9‐shot acquisition (scan time = 18 s) and 12/4 for the 15‐shot acquisition (scan time = 30 s).

An additional axial head–neck dataset was acquired using a 15‐shot gradient‐echo WFS‐EPTI sequence. The acquisition parameters were as follows: resolution = 1 × 1 × 3 mm^3^, acquisition matrix = 204 × 180, 10 slices, flip angle = 90°, TR = 800 ms, TE range = 11.8–63.4 ms, ETL = 44, *R*_*seg*_/*R*_*PE*_ = 12/4, no fat saturation, scan time = 30 s.

#### 3.2.3 Experiment 3: Abdominal Imaging With Auto‐Calibrated WFS‐EPTI

Abdominal images were acquired using auto‐calibrated WFS‐EPTI with a 16‐channel body matrix coil. A breath‐hold acquisition method was used. The acquisition parameters were as follows: resolution = 1 × 1 × 3 mm^3^, acquisition matrix = 328 × 180, no fat saturation. For gradient‐echo: flip angle = 30°, TR = 340 ms, TE range = 14.2–61 ms, 5 slices, ETL = 40; for spin‐echo, flip angle = 90°, TR = 700 ms, TE range = 37.4–81.8 ms, 7 slices, ETL = 38. The auto‐calibrated region has 18 fully‐sampled central lines and 14 moderately accelerated adjacent lines (acceleration factor *R*_*PE*_ = 3), resulting in a total of 32 lines acquired. The peripheral highly accelerated regions were sampled using 10 shots with an acceleration factor of *R*_*seg*_/*R*_*PE*_ = 12/4. The scan time was 14.3 s for gradient‐echo and 29.4 s for spin‐echo. Conventional EPI images with and without fat saturation (GRAPPA = 4, 10 averages) were also acquired for comparison.

To assess the robustness of externally calibrated WFS‐EPTI in abdominal imaging, a gradient‐echo and a spin‐echo WFS‐EPTI data were acquired additionally. The scan‐time was 6.8 s for the gradient‐echo (20‐shot) and 11.2 s for the spin‐echo imaging (16‐shot). All the other acquisition parameters were kept the same as in the auto‐calibrated WFS‐EPTI experiments. For reconstruction, the auto‐calibration central *k*‐space data from the corresponding auto‐calibrated WFS‐EPTI acquired in another breath‐hold served as calibration data for the corresponding gradient‐echo and the spin‐echo data, respectively.

## 4 Results

**FIGURE 4:**
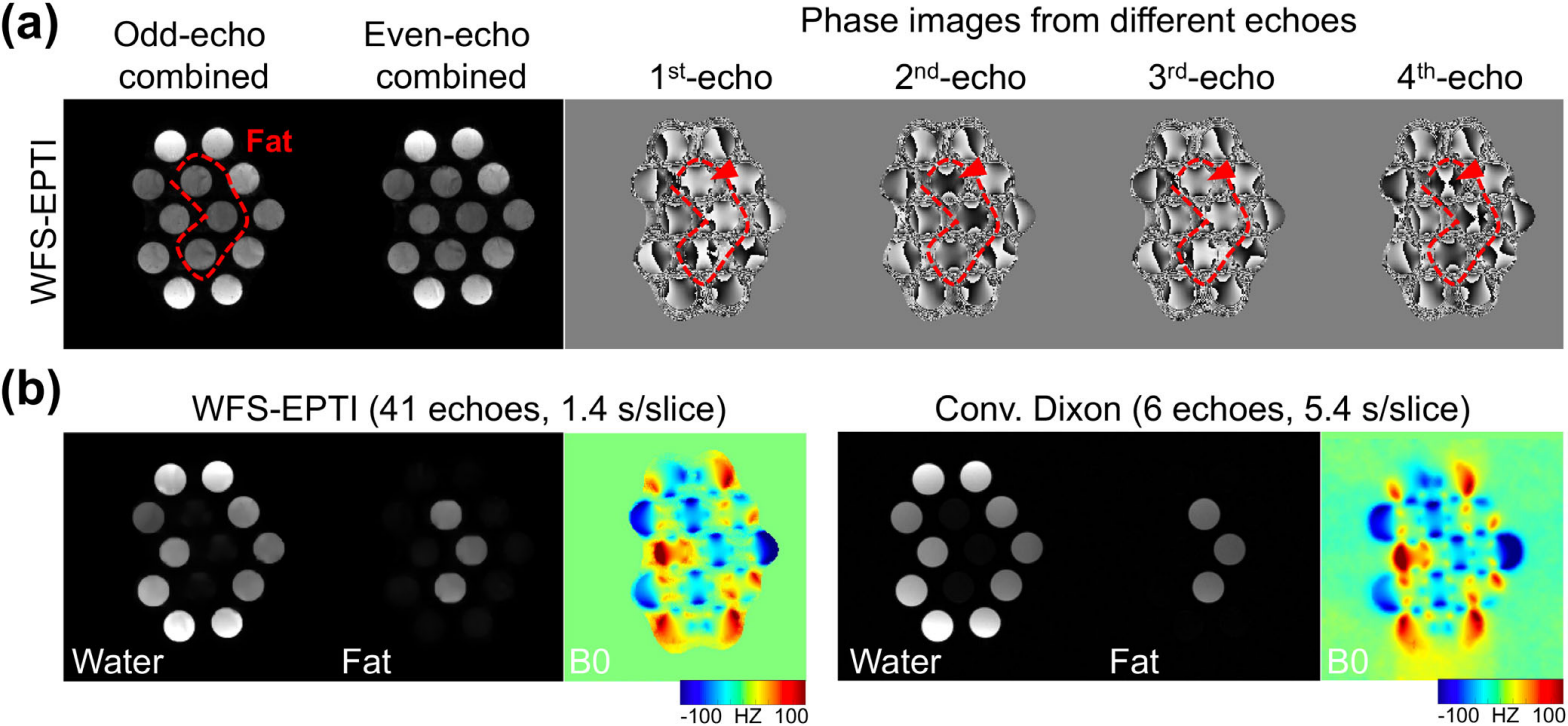
Water‐fat phantom evaluation. (a) Odd‐ and even‐echo combined images along with phase images from the first four echoes, acquired using gradient‐echo WFS‐EPTI with 17 shots. The 3 tubes within the red‐dashed line contain fat, while the remaining tubes contain water. The phase of fat evolves much more rapidly than that of water, as indicated by the red arrows in the phase images. (b) Water‐only and fat‐only images, as well as B0 maps, estimated from the proposed WFS‐EPTI (left) and a lengthier conventional Dixon method (right). Both methods achieved clear separation of fat and water signals in the phantom under relatively strong B0 inhomogeneity (up to 100 Hz).

In the phantom experiment, the odd and even‐echo images acquired with WFS‐EPTI can be successfully reconstructed with minimal fat artifacts (Figure 4a). Note that the phases of fat (pointed by the arrows) evolve faster than that of water but remain relatively stable within the odd echoes or within the even echoes, as expected. The water/fat images and B0 maps calculated from WFS‐EPTI were comparable to those obtained with the much longer conventional GRE acquisition (Figure 4b), demonstrating the feasibility and efficacy of the proposed method in water/fat separation and its superior acquisition efficiency (14 s vs. 54 s).

**FIGURE 5:**
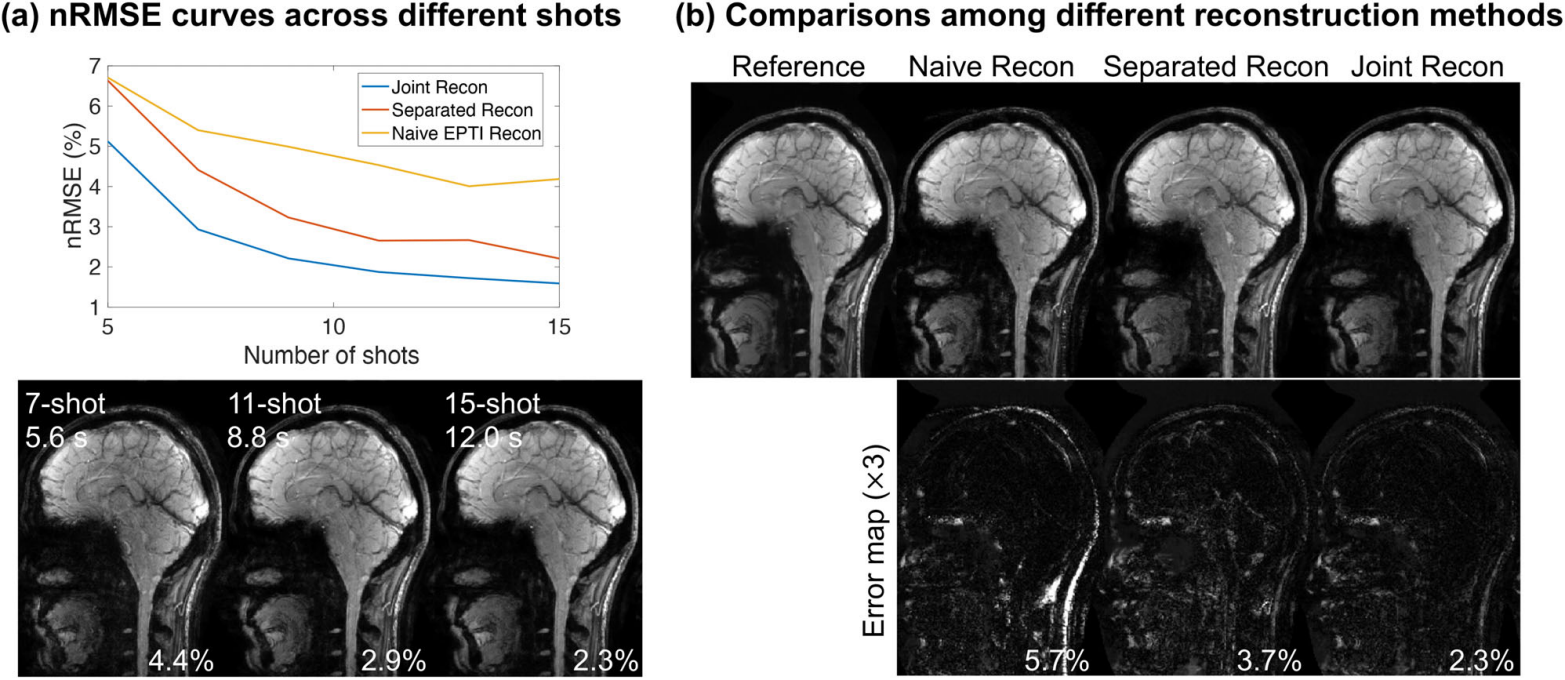
Retrospective undersampling experiment for comparison of naive EPTI reconstruction, separately and jointly reconstructed WFS‐EPTI. (a) The nRMSE curves across different shot numbers illustrate the superior performance of the joint reconstruction method. The proposed WFS‐EPTI can be well reconstructed under different shot numbers. The corresponding acquisition times for different shot numbers (7, 11, and 15 shots) are also shown. (b) Example reconstructed images from the 15‐shot condition. The joint reconstruction achieves the lowest reconstruction errors, as demonstrated by the error maps (scaled by a factor of 3) and the normalized root mean squared error (nRMSE) values displayed at the bottom‐right corners.

The performances of the three reconstruction approaches—conventional naive EPTI reconstruction, the separate and the joint WFS‐EPTI reconstruction—under different acceleration factors were evaluated by retrospectively undersampling the fully sampled data. The nRMSE curves indicate that the joint reconstruction approach consistently yielded the lowest error across all acceleration factors (Figure 5a). In addition, as expected, increasing the number of shots (i.e., lowering the acceleration) enhanced image quality, as demonstrated by the representative 7, 11, and 15‐shot examples. Compared with fully sampled reference images and their corresponding error maps (Figure 5b), both the separated and the joint WFS‐EPTI reconstruction recovered fat signals more accurately than the naive method, and the joint reconstruction outperformed the separated reconstruction, especially in brain regions containing mainly water signals.

**FIGURE 6:**
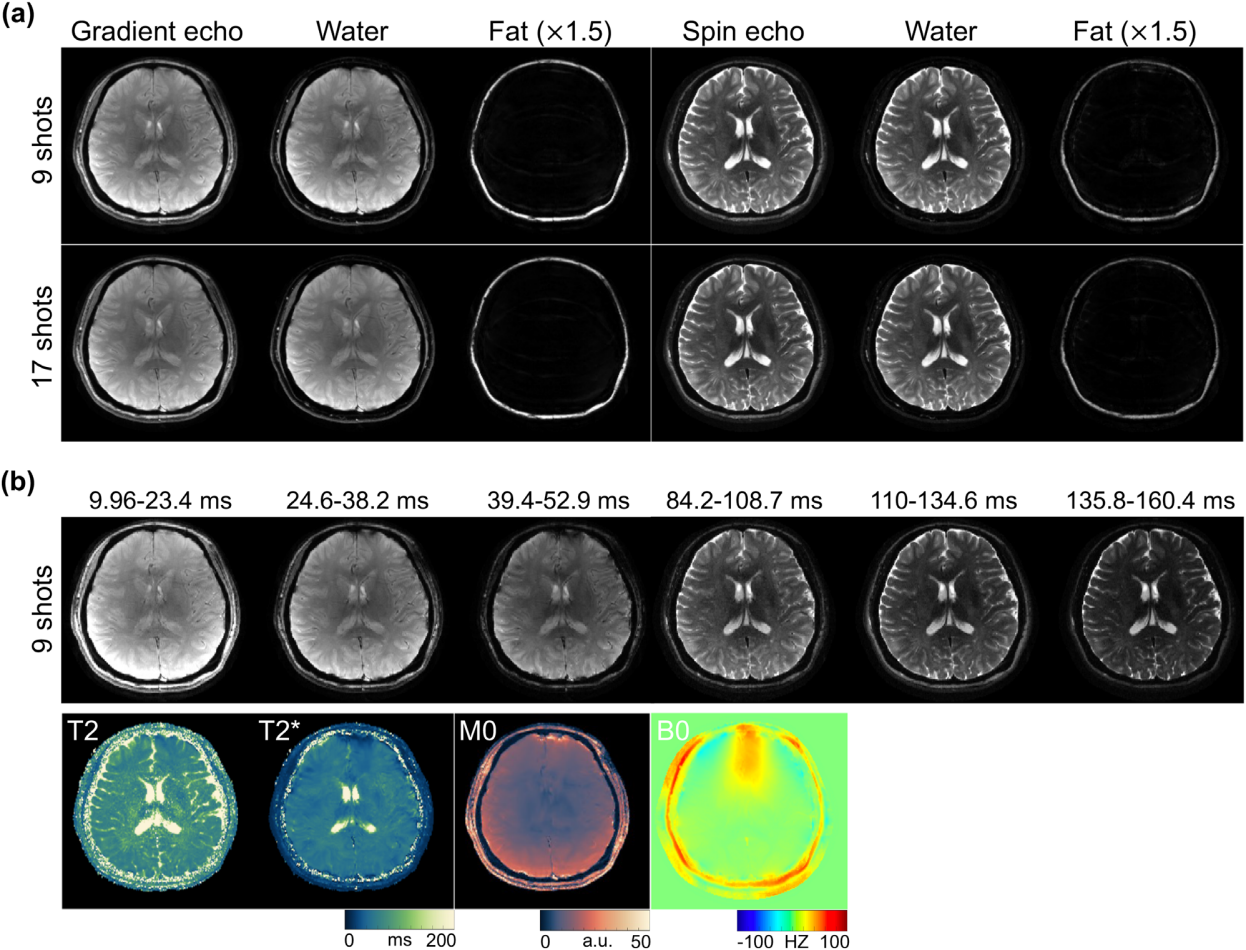
In vivo human brain imaging results obtained with the proposed WFS‐EPTI technique. (a) Echo‐combined images, as well as water‐only and fat‐only images, acquired using 9‐shot and 17‐shot GESE WFS‐EPTI without applying any fat saturation. (b) Multi‐contrast images and quantitative maps obtained from the 9‐shot GESE WFS‐EPTI, including echo‐combined multi‐contrast images at different echo times, T2, T2*, M0, and B0 maps.

Figure 6a shows the brain imaging results from a 9‐shot and a 17‐shot GESE WFS‐EPTI sequence with high image quality. Water/fat signals were successfully separated, and multi‐contrast images and quantitative maps (T2, T2*, M0, B0) were obtained, as shown for the 9‐shot WFS‐EPTI (Figure 6b) acquired in 18 s.

**FIGURE 7:**
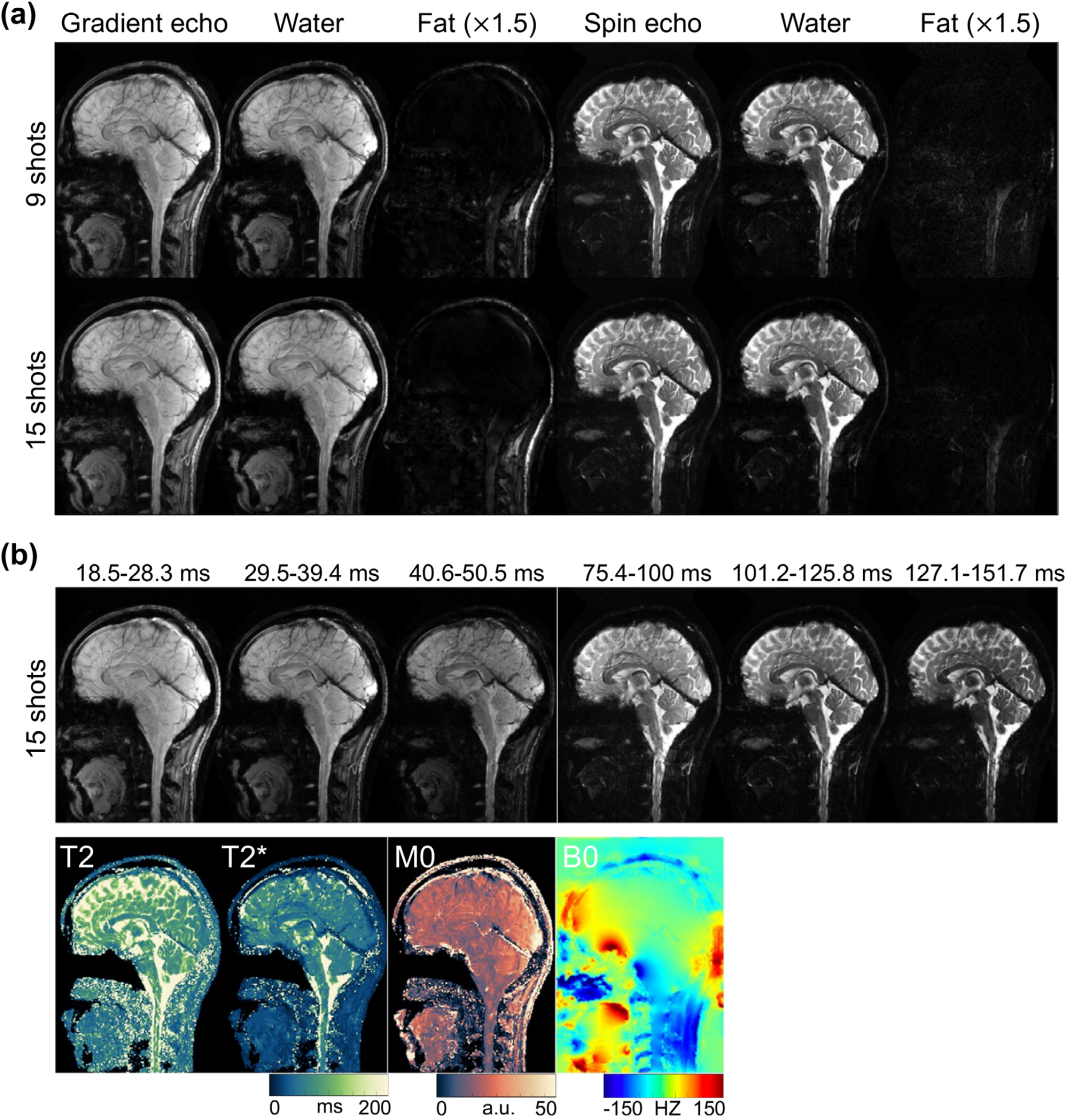
In vivo human head–neck imaging results obtained with the proposed WFS‐EPTI technique. (a) Echo‐combined images, as well as water‐only and fat‐only images, acquired using 9‐shot and 15‐shot GESE WFS‐EPTI in a sagittal acquisition without fat saturation. (b) Multi‐contrast images and quantitative maps obtained from the 15‐shot GESE WFS‐EPTI, including echo‐combined multi‐contrast images at different echo times, T2, T2*, M0, and B0 maps. The estimated B0 maps show the stronger field inhomogeneity of the head–neck regions than brain.

**FIGURE 8:**
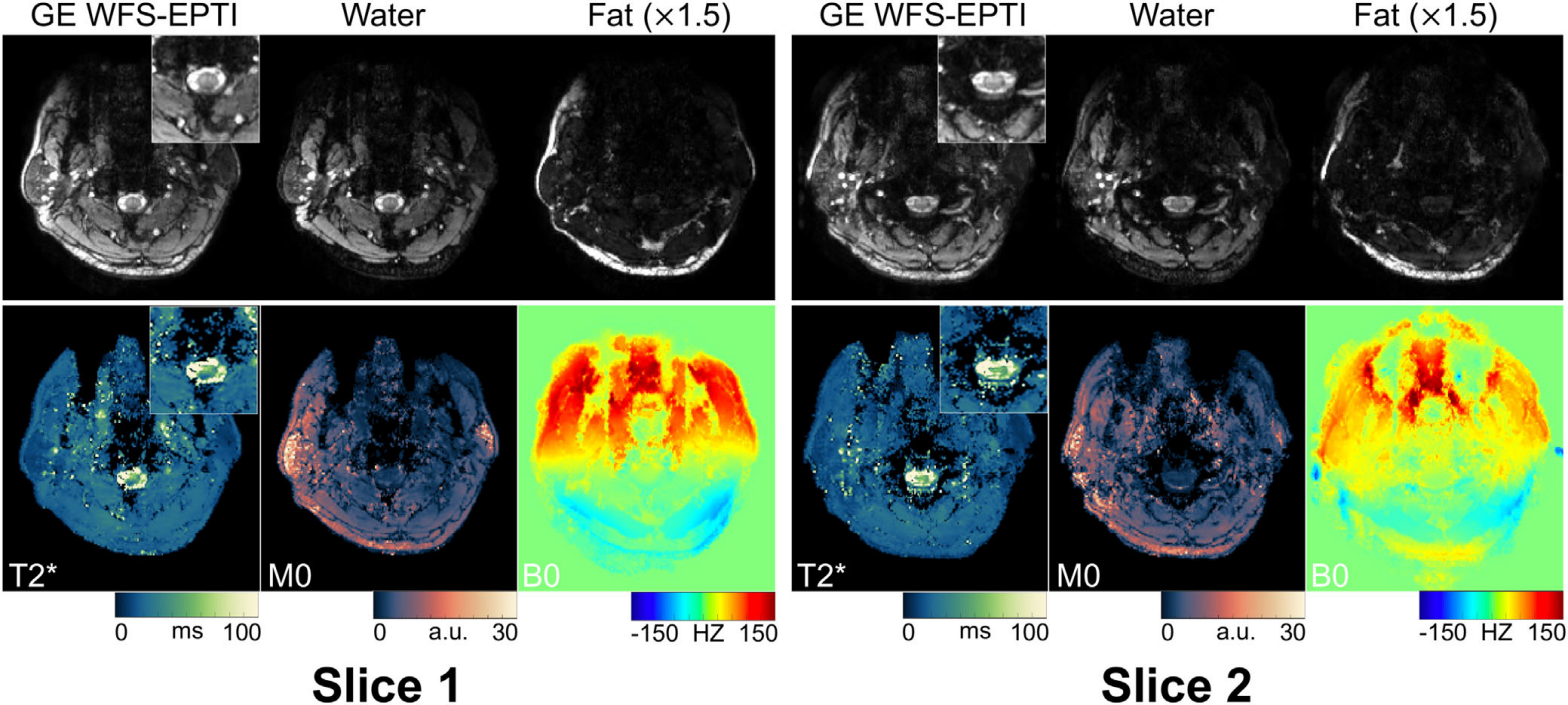
In vivo human neck imaging results obtained with the proposed WFS‐EPTI technique. Echo‐combined images, as well as water‐only and fat‐only images from two different slices, acquired using 15‐shot gradient‐echo WFS‐EPTI without fat saturation. Corresponding quantitative maps, including T2*, M0, and B0 maps, are also shown. Insets show zoomed‐in views of the spinal cord regions.

When imaging regions outside the brain, such as the neck and abdomen, field inhomogeneity and fat signals can become increasingly prominent. For head–neck imaging as shown in Figure 7a (sagittal slices), WFS‐EPTI with 9‐shot and 15‐shot GESE acquisition both demonstrated high image quality and successful water/fat separation. Example multi‐contrast images and quantitative maps (T2, T2*, M0, B0) are shown in Figure 7b which were acquired in 30 s. Figure 8 presents two axial head–neck images acquired with a 15‐shot gradient‐echo WFS‐EPTI sequence. The reconstructed images, the corresponding water‐ and fat‐only images, and the quantitative maps (T2*, M0, B0) collectively demonstrate the efficacy of the proposed approach. Insets with zoomed‐in views of the spinal cord highlight the distortion‐free advantage of EPTI technique in regions affected by severe B0 inhomogeneity.

**FIGURE 9:**
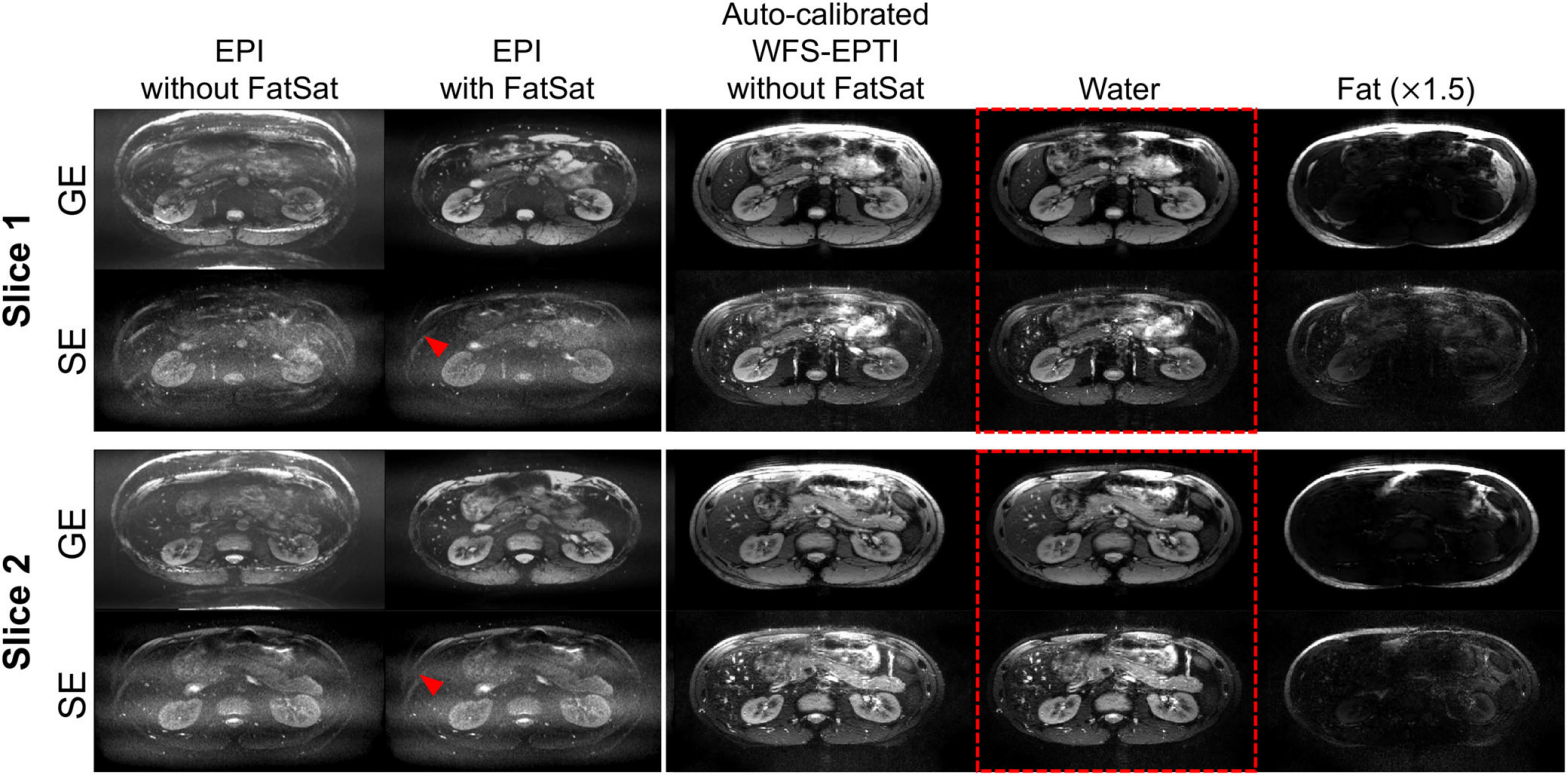
In vivo human abdominal imaging results obtained using the proposed auto‐calibrated WFS‐EPTI technique. EPI images without and with fat saturation, and auto‐calibrated WFS‐EPTI's (without fat saturation) echo‐combined images, water‐only and fat‐only images are shown for two slices with both gradient and spin echo contrast. Conventional EPI images demonstrated issues such as geometric distortion and residual fat artifacts even with fat saturation. In contrast, the proposed approach, without fat saturation, provides high‐resolution images with minimal fat artifacts and improved image quality.

**FIGURE 10:**
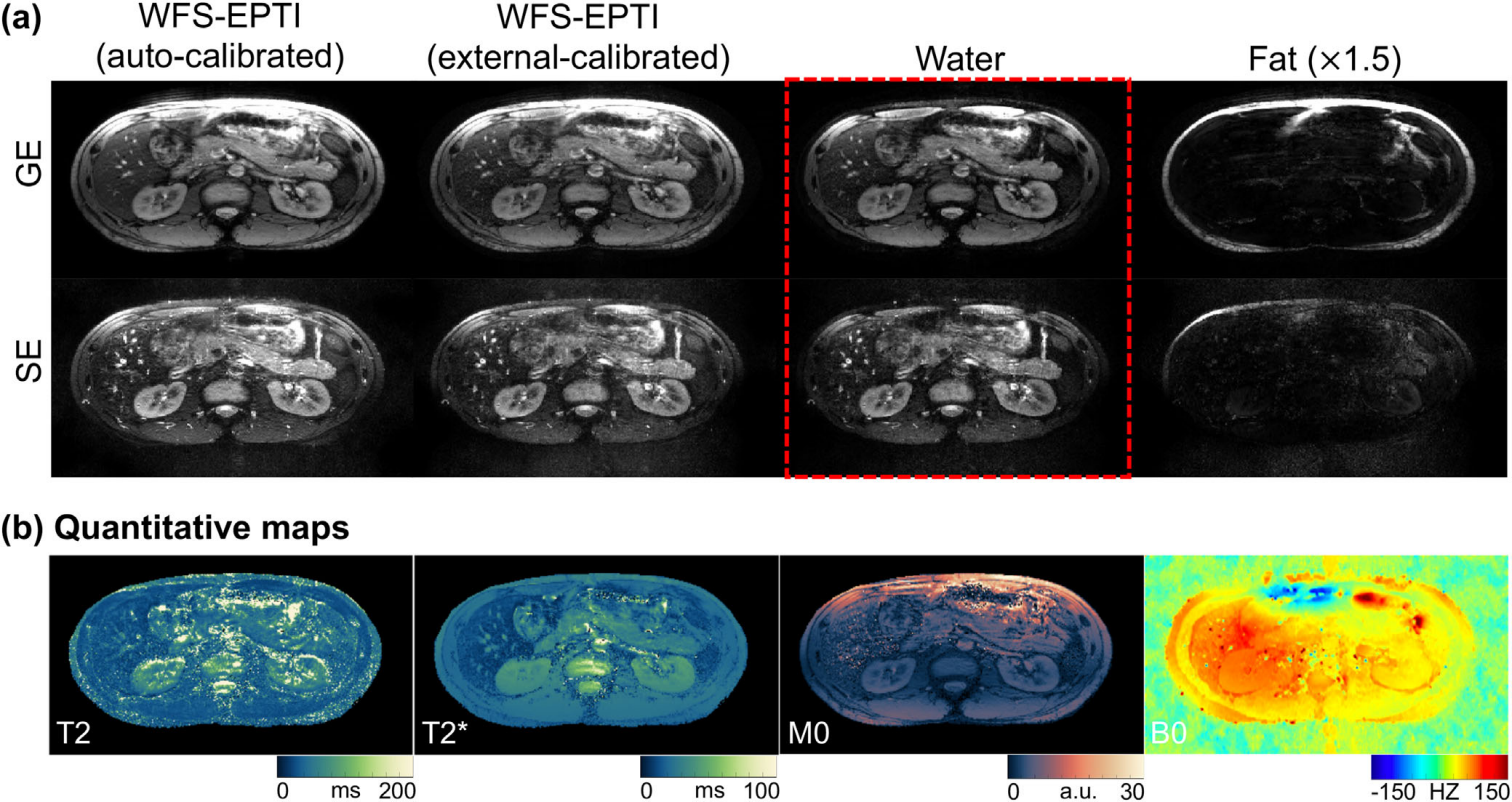
In vivo human abdominal imaging results obtained using auto‐calibrated and external‐calibrated WFS‐EPTI. (a) Echo combined images, along with corresponding water‐only and fat‐only images, acquired using WFS‐EPTI with external calibration, are presented for a representative slice. The scan time was 6.8 s for gradient‐echo and 11.2 s for spin‐echo. Corresponding images acquired using the auto‐calibrated version of WFS‐EPTI are also shown as the reference on the left, demonstrating the comparable image quality achieved by the externally calibrated WFS‐EPTI. (b) Quantitative maps derived from the abdominal WFS‐EPTI data, including T2, T2*, M0, and B0 maps.

Abdominal imaging results obtained by WFS‐EPTI with GE and SE acquisition are shown in Figures 9 and 10. Figure 9 compares abdominal images acquired using conventional EPI and auto‐calibrated WFS‐EPTI. Images acquired with conventional EPI without fat saturation show severe chemical‐shift artifacts, low SNR, and geometric distortion. Even with fat saturation, residual chemical‐shift artifacts persisted as highlighted by the arrows. In contrast, the proposed WFS‐EPTI produced distortion‐free images without noticeable chemical‐shift artifacts even without applying fat saturation. The fat signals can also be successfully separated, resulting in water‐only images with high fidelity. Results from 3 additional subjects are provided in Figure S3.

Figure 10 shows the WFS‐EPTI results when an external calibration scan from another breath hold was used (results from 3 additional subjects are provided in Figure S4). Specifically, using the external calibration data can yield image quality comparable to that of auto‐calibrated WFS‐EPTI (Figure 10a) while reducing scan time, providing greater flexibility for accelerations. Quantitative maps (T2, T2*, M0, B0) obtained from the GESE images further show the image quality and efficiency provided by the proposed method for abdominal imaging.

## 5 Discussion

EPTI technique addresses EPI's distortion and T2/T2*‐blurring artifacts and can produce distortion‐free multi‐echo/multi‐contrast images with high efficiency, making it a practical, ultra‐fast acquisition for a wide range of MRI applications. However, the challenge of fat signals needs to be addressed for its broader applications in body imaging where insufficient fat saturation often occurs. The proposed WFS‐EPTI method enables water/fat separation by leveraging the intrinsic multi‐echo data from EPTI and exploiting a novel strategy in spatiotemporal encoding and reconstruction, while retaining EPTI's key advantages—high resolution, distortion‐free imaging, and fast acquisition. The efficacy of the proposed approach, using gradient‐echo and/or spin‐echo contrasts, was demonstrated in both phantom and in vivo experiments across multiple body regions from brain to neck and abdomen.

The multi‐echo data of EPTI readout intrinsically encodes the phase of water and fat signals, making it well‐suitable for efficient water/fat separation. While model‐based reconstruction has been used to model water/fat signals in multi‐echo acquisitions [55], enforcing strict models can be vulnerable to deviations in signal evolution from the assumed model. Furthermore, the high non‐convexity of model‐based reconstruction makes it sensitive to the initial guess, which is often obtained from low‐resolution images—for example, derived from variable density *k*‐space sampling trajectories, such as radial sampling [56]—thereby limiting the achievable acceleration. Zhou et al. recently proposed a water/fat separated approach based on EPTI using spatiotemporal joint reconstruction aided by prior water/fat signal masks [57]. In this study, we use a subspace approach combined with regular EPTI acquisition that avoids explicit model enforcement and prior water/fat image constraints, providing greater flexibility and robustness to multi‐compartmental signal evolutions while enabling high acceleration capability [44]. However, the rapidly varying phase evolution of fat across echoes due to chemical shift compromises the low‐rank properties underpinning the subspace approach, posing a challenge for accurate reconstruction under high accelerations.

To address this, the concept of DIXON method was incorporated into WFS‐EPTI readout by appropriately scheduling the TE and echo spacing, with the goal of controlling and modulating fat phase evolution across the readout into a more uniform, consistent manner. This allows the fat signal evolution to exhibit consistent phase behavior within each echo group (e.g., in‐phase for all odd echoes and out‐of‐phase for all even echoes) and be effectively represented in the reconstruction, e.g., using a smaller number of subspace basis. Then, to prevent potential SNR and reconstruction conditioning loss from the reduced data amount when splitting into groups (e.g., odd and even echoes), a joint subspace reconstruction is proposed to leverage both datasets. As expected, the joint reconstruction approach achieved higher image quality than the separate reconstruction (Figure 5), particularly in brain regions dominated by water signals, where the odd‐ and even‐echo datasets share substantial information that can be effectively exploited via the joint constraints.

In this study, we conservatively used low acceleration factors (high numbers of shots), allowable within a breath‐hold duration, because the focus of this study was anatomical and quantitative imaging, which places less emphasis on high temporal resolution compared to applications like functional or diffusion MRI. Second, fat signals were addressed entirely through the encoding and reconstruction process, without relying on any fat saturation pulses, avoiding deadtime and SNR loss. Using more shots also helps compensate for the reduced data amount when splitting into echo groups to address fat‐related phase evolution in WFS‐EPTI.

The echo‐spacing phase scheduling and reconstruction strategies exploited in this study can be generalized to other studies, such as multi‐echo reconstruction using subspace approaches [33, 43, 44] or *k*‐space based methods (e.g., B0‐GRAPPA [58]), where data groups with inter‐group phase differences but intra‐group consistency may be modeled using separate basis, low‐rank structures, or kernels to improve fidelity, while jointly regularized to preserve conditioning. It can also be adapted for conventional EPI reconstruction, where odd and even *k*‐space lines may be echo‐spacing scheduled and reconstructed separately to reduce associated artifacts.

After subspace reconstruction, a *k*‐space domain algorithm [49] was employed for water/fat separation to account for acquisition timing differences between the readout samples of odd and even echoes. This approach helps correct potential chemical‐shift misregistration of water and fat signals along the readout direction caused by the bipolar readout in echo‐planar acquisition. Given the high readout bandwidth (∼1.5 kHz/pixel in EPTI acquisition) used in this study, no obvious chemical shift along readout dimension was observed in our data, suggesting that conventional image domain water/fat separation algorithms [23, 47, 48] can also be applicable. However, for future applications with lower readout bandwidth, the *k*‐space based method may offer added benefits. The optimal choice of echo time shifts has been discussed in previous works [19, 59]. Asymmetric echoes are generally recommended for reliable water/fat separation. Nevertheless, to enable subspace reconstruction for fast acquisition, the time shifts (i.e., ESP) in WFS‐EPTI were constrained to achieve in‐phase and out‐of‐phase conditions for odd‐ and even‐echoes. Thanks to the redundant data provided by EPTI acquisition (typically ∼30–40 echoes were acquired), reliable water/fat separation was achieved across different imaging protocols and organs in this study, demonstrating the robustness of the proposed approach. Extending the current 2‐point WFS‐EPTI method to a 3‐point or multi‐point strategy could further enhance water/fat separation performance.

The echo‐spacing phase scheduling strategy assumes that the odd and even echoes correspond to in‐phase and out‐of‐phase conditions, respectively. In practice, due to the dominant contribution of the main fat spectral peak, this in‐phase/out‐of‐phase assumption remains reasonably valid—an observation further supported by the widespread clinical use of the two‐point DIXON method [60]. To further improve robustness, here we discuss both existing strategies used in this study and future approaches to address potential deviations. First, inaccuracies or phase deviations arising from limitations in protocol parameter precision or potential deviations in the actual chemical shift from its assumed value caused by temperature or other factors—particularly in phantom imaging—are relatively minor and can be accounted for by incorporating them into the basis generation process. Second, chemical shifts from other fat components beyond the main spectral peak [51, 52] can lead to more substantial phase deviations. Figure S1 illustrates that a multi‐peak fat spectrum model necessitates more complex bases to represent the signal, especially when the relative amplitudes of the multiple spectral peaks of fat are *not* known a priori during subspace basis generation. To address these, WFS‐EPTI was designed with: (i) a complex‐valued subspace reconstruction with complex basis capable of capturing small phase deviations; and (ii) a data‐driven basis generation process, along with the auto‐calibrated technique, which can capture data‐specific signal fluctuations resulting from imperfect in‐phase/out‐of‐phase conditions rather than relying on bases generated from predefined multi‐peak fat spectrum simulation. Figure S2 demonstrates how imperfect in‐phase/out‐of‐phase conditions induced by the multi‐peak fat spectrum biased the reconstruction performance using simulated bases assuming perfect in‐phase/out‐of‐phase conditions, and that the proposed data‐driven basis can mitigate this bias. Residual artifacts observed in the fat images are likely attributable to the spectral complexity of fat signals still not being fully represented; future work will address this by further optimization of bases generation and selection. Overall, experiments conducted across multiple anatomical regions in this study demonstrated the robustness of the proposed subspace reconstruction.

As a proof‐of‐concept study to demonstrate the feasibility of the proposed encoding and subspace reconstruction for addressing fat signals, we did not particularly optimize the acquisition speed. For example, we have not yet integrated simultaneous multi‐slice (SMS) [61, 62] in our 2D acquisition, nor have we directly used 3D‐EPTI acquisition [33], both of which can substantially increase efficiency. Further optimization of the sampling strategy to reduce the number of shots, together with other acceleration techniques, should further reduce the acquisition time. Future integration with 3D EPTI acquisition could facilitate extensions to other contrasts such as T1‐weighted or quantitative T1 imaging. For abdominal imaging applications, a breath‐hold scheme was used to account for respiratory motion in this study. Future work will also adopt strategies to address motion more efficiently, such as respiratory triggering, inter‐shot motion correction [36, 63], or retrospective data sorting/binning and motion‐robust encodings [64] for free‐breathing dynamic imaging. As an alternative scheme, the external‐calibrated WFS‐EPTI was performed to demonstrate the feasibility of using an externally acquired calibration to reduce breath‐hold duration (Figure 10 and Figure S4). Good performance was achieved in three of the total four subjects tested, while artifacts were observed in one subject due to severe motion‐induced mismatch between the calibration‐derived sensitivity and B0 maps and the imaging scans across different breath‐hold states, which will be addressed in future work for external calibration. Finally, besides the WFS images, preliminary results of quantitative mapping were presented in this study. Future work will further validate the accuracy and precision of the calculated maps across more scenarios.

## 6 Conclusions

A novel WFS‐EPTI approach was proposed in this study, which effectively resolves fat signals in the subspace reconstruction of conventional EPTI and provides high‐resolution, distortion‐free multi‐contrast images. By leveraging an echo‐spacing‐scheduled, spatiotemporal‐encoded acquisition, together with a jointly regularized subspace reconstruction, the technique enables effective water–fat separation and allows EPTI to suppress fat‐related signals and artifacts without the need for additional fat saturation preparations. Both phantom and in vivo experiments across multiple body regions, from brain to neck and abdomen, demonstrated the efficacy of the proposed approach.

## Funding

This work was supported by National Institutes of Health, P41‐EB030006, R00‐AG083056, R01‐EB019437, R01‐EB036507, U24‐NS129893.

## Supporting information


**Figure S1:** Signal evolution and subspace basis for a single‐peak fat spectrum (a) and a multi‐peak fat spectrum (b) for odd and even echoes. The multi‐peak model includes fat components with chemical shifts at 3.80, 3.40, 2.60, 1.94, 0.39, −0.60 ppm, while the single‐peak model only considers the main peak at 3.40 ppm. Note that, during subspace basis generation, the top panel uses fixed relative amplitudes of 0.087, 0.693, 0.128, 0.004, 0.039, 0.04 for the multi‐peak fat components, whereas the bottom panel shows the results when no constraints were imposed on the relative amplitudes of the fat components. The echo time and echo‐spacing were chosen such that in‐phase/out‐of‐phase condition can be achieved for the main fat peak. The signal evolutions were simulated with 44 echoes ranging from 12 to 63.6 ms, with an echo spacing of 1.2 ms. The left column presents simulated real and imaginary of water (red) and fat (blue) signals across echo times. The middle column illustrates the first three temporal basis components derived from singular value decomposition (SVD). The right column shows the corresponding singular value spectra (coefficients), with the number of significant basis components indicated by the red dashed lines.
**Figure S2:** WFS‐EPTI reconstructions simulated with a numerical water/fat phantom. Data were simulated using a single‐peak fat spectrum (a) and a multi‐peak fat spectrum (b). Specifically, the data simulated with the 6‐peak fat model assumed relative amplitudes of 0.087, 0.693, 0.128, 0.004, 0.039, and 0.04, respectively. The phantom's proton density, proton density fat fraction (PDFF), and T2* values were used to generate fully sampled WFS‐EPTI data, along with simulated B0 maps. The data were simulated with 44 echoes ranging from 12 to 63.6 ms, with an echo spacing of 1.2 ms. The data were reconstructed using either simulated basis assuming perfect in‐phase/out‐of‐phase conditions (i.e., no phase modulation due to chemical‐shift effects, with separate bases for odd and even echoes, respectively) or data‐driven basis (as obtained in auto‐calibrated WFS‐EPTI). Echo‐combined images, and water/fat‐separated images are shown, together with error maps of the reconstructed images relative to the ground‐truth images. Five acceleration factors were simulated, corresponding to acquisitions using 7, 9, 11, 13, and 15 shots. The results for the 15‐shot case are shown in (a) and (b). The corresponding nRMSE curves under different conditions are shown in (c).
**Figure S3:** In vivo human abdominal imaging results obtained using the proposed auto‐calibrated WFS‐EPTI technique in 3 additional subjects. Data were acquired using the same acquisition matrix but different field‐of‐views tailored to individual body sizes (subject 2: 400 × 280 mm^2^; subject 3: 344 × 240 mm^2^; subject 4: 344 × 240 mm^2^). Echo‐combined, water‐only and fat‐only images reconstructed from auto‐calibrated WFS‐EPTI without fat saturation are shown with both gradient and spin echo contrast. Auto‐calibrated WFS‐EPTI images acquired with fat saturation are also provided as the references. The proposed approach yields water‐only images with minimal fat signal artifacts and image quality comparable to that of the data acquired with fat saturation. Note that images with different contrasts were acquired in separate breath‐holds and therefore may not correspond to the same locations in each subject. Artifacts due to large vessels can be observed in Subject 3.
**Figure S4:** In vivo human abdominal imaging results obtained using auto‐calibrated and external‐calibrated WFS‐EPTI in 3 additional subjects. Data were acquired using the same acquisition matrix but different field‐of‐views tailored to individual body sizes (subject 2: 400 × 280 mm^2^; subject 3: 344 × 240 mm^2^; subject 4: 344 × 240 mm^2^). Echo combined images, along with corresponding water‐only and fat‐only images, acquired using WFS‐EPTI with external calibration, are presented. Corresponding images acquired using the auto‐calibrated version of WFS‐EPTI are also shown as the reference on the left. External‐calibrated WFS‐EPTI shows lower SNR compared with auto‐calibrated ones due to less data samples, as expected. Note that images with different contrasts were acquired in separate breath‐holds and therefore may not correspond to the same locations in each subject. WFS‐EPTI with external calibration showed good performance in three out of the total four subjects, while one subject exhibited artifacts due to severe motion‐induced mismatch between the calibration and image scans. Future work will address the vulnerability of external‐calibration to motion‐related misregistration of images anatomical structures across different breath‐hold states.

## Data Availability

The data that support the findings of this study are available on request from the corresponding author. The data are not publicly available due to privacy or ethical restrictions.

## References

[1] F. Schmitt , M. K. Stehling , and R. Turner , Echo‐Planar Imaging (Springer Berlin Heidelberg, 1998).

[2] J. Burakiewicz , G. D. Charles Edwards , V. Goh , and T. Schaeffter , “Water‐Fat Separation in Diffusion‐Weighted EPI Using an IDEAL Approach With Image Navigator,” Magnetic Resonance in Medicine 73, no. 3 (2015): 964–972. 24723244 10.1002/mrm.25191

[3] D. Hernando , D. C. Karampinos , K. F. King , et al., “Removal of Olefinic Fat Chemical Shift Artifact in Diffusion MRI,” Magnetic Resonance in Medicine 65, no. 3 (2011): 692–701. 21337402 10.1002/mrm.22670 PMC3069507

[4] J. Hansmann , D. Hernando , and S. B. Reeder , “Fat Confounds the Observed Apparent Diffusion Coefficient in Patients With Hepatic Steatosis,” Magnetic Resonance in Medicine 69, no. 2 (2013): 545–552. 23161434 10.1002/mrm.24535 PMC3556190

[5] S. C. Partridge , L. Singer , R. Sun , et al., “Diffusion‐Weighted MRI: Influence of Intravoxel Fat Signal and Breast Density on Breast Tumor Conspicuity and Apparent Diffusion Coefficient Measurements,” Magnetic Resonance Imaging 29, no. 9 (2011): 1215–1221. 21920686 10.1016/j.mri.2011.07.024 PMC3199288

[6] M. Maehara , K. Ikeda , H. Kurokawa , et al., “Diffusion‐Weighted Echo‐Planar Imaging of the Head and Neck Using 3‐T MRI: Investigation Into the Usefulness of Liquid Perfluorocarbon Pads and Choice of Optimal Fat Suppression Method,” Magnetic Resonance Imaging 32, no. 5 (2014): 440–445. 24582547 10.1016/j.mri.2014.01.011

[7] P. Murtz , M. Tsesarskiy , A. Kowal , et al., “Diffusion‐Weighted Magnetic Resonance Imaging of Breast Lesions: The Influence of Different Fat‐Suppression Techniques on Quantitative Measurements and Their Reproducibility,” European Radiology 24, no. 10 (2014): 2540–2551. 24898097 10.1007/s00330-014-3235-5

[8] A. Haase , J. Frahm , W. Hanicke , and D. Matthaei , “1H NMR Chemical Shift Selective (CHESS) Imaging,” Physics in Medicine and Biology 30, no. 4 (1985): 341–344. 4001160 10.1088/0031-9155/30/4/008

[9] C. Oh , “Selective Partial Inversion Recovery (SPIR) in Steady State for Selective Saturation Magnetic Resonance Imaging (MRI),” 1988.

[10] E. Kaldoudi , S. C. Williams , G. J. Barker , and P. S. Tofts , “A Chemical Shift Selective Inversion Recovery Sequence for Fat‐Suppressed MRI: Theory and Experimental Validation,” Magnetic Resonance Imaging 11, no. 3 (1993): 341–355. 8505868 10.1016/0730-725x(93)90067-n

[11] G. Krinsky , N. M. Rofsky , and J. C. Weinreb , “Nonspecificity of Short Inversion Time Inversion Recovery (STIR) as a Technique of Fat Suppression: Pitfalls in Image Interpretation,” AJR American Journal of Roentgenology 166, no. 3 (1996): 523–526. 8623620 10.2214/ajr.166.3.8623620

[12] P. A. Baltzer , M. Benndorf , M. Dietzel , M. Gajda , O. Camara , and W. A. Kaiser , “Sensitivity and Specificity of Unenhanced MR Mammography (DWI Combined With T2‐Weighted TSE Imaging, ueMRM) for the Differentiation of Mass Lesions,” European Radiology 20, no. 5 (2010): 1101–1110. 19936758 10.1007/s00330-009-1654-5

[13] A. Stadlbauer , R. Bernt , S. Gruber , et al., “Diffusion‐Weighted MR Imaging With Background Body Signal Suppression (DWIBS) for the Diagnosis of Malignant and Benign Breast Lesions,” European Radiology 19, no. 10 (2009): 2349–2356. 19415286 10.1007/s00330-009-1426-2

[14] T. Kazama , K. Nasu , Y. Kuroki , S. Nawano , and H. Ito , “Comparison of Diffusion‐Weighted Images Using Short Inversion Time Inversion Recovery or Chemical Shift Selective Pulse as Fat Suppression in Patients With Breast Cancer,” Japanese Journal of Radiology 27, no. 4 (2009): 163–167. 19499306 10.1007/s11604-009-0314-7

[15] V. Vandecaveye , F. De Keyzer , P. Dirix , M. Lambrecht , S. Nuyts , and R. Hermans , “Applications of Diffusion‐Weighted Magnetic Resonance Imaging in Head and Neck Squamous Cell Carcinoma,” Neuroradiology 52, no. 9 (2010): 773–784. 20631998 10.1007/s00234-010-0743-0

[16] Y. Bae , B. Choi , H.‐K. Jeong , L. Sunwoo , C. Jung , and J. Kim , “Diffusion‐Weighted Imaging of the Head and Neck: Influence of Fat‐Suppression Technique and Multishot 2D Navigated Interleaved Acquisitions,” American Journal of Neuroradiology 39, no. 1 (2018): 145–150. 29122759 10.3174/ajnr.A5426 PMC7410699

[17] W. T. Dixon , “Simple Proton Spectroscopic Imaging,” Radiology 153, no. 1 (1984): 189–194. 6089263 10.1148/radiology.153.1.6089263

[18] G. Glover and E. Schneider , “Three‐Point Dixon Technique for True Water/Fat Decomposition With B0 Inhomogeneity Correction,” Magnetic Resonance in Medicine 18, no. 2 (1991): 371–383. 2046518 10.1002/mrm.1910180211

[19] S. B. Reeder , A. R. Pineda , Z. Wen , et al., “Iterative Decomposition of Water and Fat With Echo Asymmetry and Least‐Squares Estimation (IDEAL): Application With Fast Spin‐Echo Imaging,” Magnetic Resonance in Medicine 54, no. 3 (2005): 636–644. 16092103 10.1002/mrm.20624

[20] S. B. Reeder , Z. Wen , H. Yu , et al., “Multicoil Dixon Chemical Species Separation With an Iterative Least‐Squares Estimation Method,” Magnetic Resonance in Medicine 51, no. 1 (2004): 35–45. 14705043 10.1002/mrm.10675

[21] H. Yu , S. B. Reeder , A. Shimakawa , J. H. Brittain , and N. J. Pelc , “Field Map Estimation With a Region Growing Scheme for Iterative 3‐Point Water‐Fat Decomposition,” Magnetic Resonance in Medicine 54, no. 4 (2005): 1032–1039. 16142718 10.1002/mrm.20654

[22] A. S. Soliman , J. Yuan , K. K. Vigen , J. A. White , T. M. Peters , and C. A. McKenzie , “Max‐IDEAL: A Max‐Flow Based Approach for IDEAL Water/Fat Separation,” Magnetic Resonance in Medicine 72, no. 2 (2014): 510–521. 24006275 10.1002/mrm.24923

[23] J. Tsao and Y. Jiang , “Hierarchical IDEAL: Fast, Robust, and Multiresolution Separation of Multiple Chemical Species From Multiple Echo Times,” Magnetic Resonance in Medicine 70, no. 1 (2013): 155–159. 22887356 10.1002/mrm.24441

[24] K. Butts , A. de Crespigny , J. M. Pauly , and M. Moseley , “Diffusion‐Weighted Interleaved Echo‐Planar Imaging With a Pair of Orthogonal Navigator Echoes,” Magnetic Resonance in Medicine 35, no. 5 (1996): 763–770. 8722828 10.1002/mrm.1910350518

[25] H. K. Jeong , J. C. Gore , and A. W. Anderson , “High‐Resolution Human Diffusion Tensor Imaging Using 2‐D Navigated Multishot SENSE EPI at 7 T,” Magnetic Resonance in Medicine: Official Journal of the Society of Magnetic Resonance in Medicine/Society of Magnetic Resonance in Medicine 69, no. 3 (2013): 793–802. 10.1002/mrm.24320 PMC3424313 22592941

[26] Z. Dong , F. Wang , X. Ma , et al., “Interleaved EPI Diffusion Imaging Using SPIR i T‐Based Reconstruction With Virtual Coil Compression,” Magnetic Resonance in Medicine 79, no. 3 (2018): 1525–1531. 28608411 10.1002/mrm.26768

[27] Z. Hu , Y. Wang , Z. Dong , and H. Guo , “Water/Fat Separation for Distortion‐Free EPI With Point Spread Function Encoding,” Magnetic Resonance in Medicine 82, no. 1 (2019): 251–262. 30847991 10.1002/mrm.27717

[28] Y. Dong , D. Atkinson , K. Koolstra , M. J. P. van Osch , and P. Bornert , “Chemical Shift‐Encoded Multishot EPI for Navigator‐Free Prostate DWI,” Magnetic Resonance in Medicine 93, no. 3 (2025): 1059–1076. 39402739 10.1002/mrm.30334 PMC11680737

[29] Y. Dong , K. Koolstra , Z. Li , M. Riedel , M. J. P. van Osch , and P. Bornert , “Structured Low‐Rank Reconstruction for Navigator‐Free Water/Fat Separated Multi‐Shot Diffusion‐Weighted EPI,” Magnetic Resonance in Medicine 91, no. 1 (2024): 205–220. 37753595 10.1002/mrm.29848

[30] Y. Dong , M. Riedel , K. Koolstra , M. J. P. van Osch , and P. Bornert , “Water/Fat Separation for Self‐Navigated Diffusion‐Weighted Multishot Echo‐Planar Imaging,” NMR in Biomedicine 36, no. 1 (2023): e4822. 36031585 10.1002/nbm.4822 PMC10078174

[31] Y. Dong , K. Koolstra , M. Riedel , M. J. P. van Osch , and P. Bornert , “Regularized Joint Water‐Fat Separation With B(0) Map Estimation in Image Space for 2D‐Navigated Interleaved EPI Based Diffusion MRI,” Magnetic Resonance in Medicine 86, no. 6 (2021): 3034–3051. 34255392 10.1002/mrm.28919 PMC8596522

[32] F. Wang , Z. Dong , T. G. Reese , et al., “Echo Planar Time‐Resolved Imaging (EPTI),” Magnetic Resonance in Medicine 81, no. 6 (2019): 3599–3615. 30714198 10.1002/mrm.27673 PMC6435385

[33] Z. Dong , F. Wang , T. G. Reese , B. Bilgic , and K. Setsompop , “Echo Planar Time‐Resolved Imaging With Subspace Reconstruction and Optimized Spatiotemporal Encoding,” Magnetic Resonance in Medicine 84, no. 5 (2020): 2442–2455. 32333478 10.1002/mrm.28295 PMC7402016

[34] F. Wang , Z. Dong , T. G. Reese , B. Rosen , L. L. Wald , and K. Setsompop , “3D Echo Planar Time‐Resolved Imaging (3D‐EPTI) for Ultrafast Multi‐Parametric Quantitative MRI,” NeuroImage 250 (2022): 118963. 35122969 10.1016/j.neuroimage.2022.118963 PMC8920906

[35] Z. Dong , F. Wang , K. S. Chan , et al., “Variable Flip Angle Echo Planar Time‐Resolved Imaging (vFA‐EPTI) for Fast High‐Resolution Gradient Echo Myelin Water Imaging,” NeuroImage 232 (2021): 117897. 33621694 10.1016/j.neuroimage.2021.117897 PMC8221177

[36] Z. Dong , F. Wang , and K. Setsompop , “Motion‐Corrected 3D‐EPTI With Efficient 4D Navigator Acquisition for Fast and Robust Whole‐Brain Quantitative Imaging,” Magnetic Resonance in Medicine 88, no. 3 (2022): 1112–1125. 35481604 10.1002/mrm.29277 PMC9246907

[37] Z. Dong , T. G. Reese , H. H. Lee , et al., “Romer‐EPTI: Rotating‐View Motion‐Robust Super‐Resolution EPTI for SNR‐Efficient Distortion‐Free In‐Vivo Mesoscale Diffusion MRI and Microstructure Imaging,” Magnetic Resonance in Medicine 93, no. 4 (2025): 1535–1555. 39552568 10.1002/mrm.30365 PMC11782731

[38] M. J. Fair , F. Wang , Z. Dong , T. G. Reese , and K. Setsompop , “Propeller Echo‐Planar Time‐Resolved Imaging With Dynamic Encoding (PEPTIDE),” Magnetic Resonance in Medicine: Official Journal of the Society of Magnetic Resonance in Medicine/Society of Magnetic Resonance in Medicine 83, no. 6 (2020): 2124–2137. 10.1002/mrm.28071 PMC7047547 31703154

[39] Z. Dong , F. Wang , L. Wald , and K. Setsompop , “SNR‐Efficient Distortion‐Free Diffusion Relaxometry Imaging Using Accelerated Echo‐Train Shifted Echo‐Planar Time‐Resolving Imaging (ACE‐EPTI),” Magnetic Resonance in Medicine 88, no. 1 (2022): 164–179. 35225368 10.1002/mrm.29198

[40] Z. Dong , L. L. Wald , J. R. Polimeni , and F. Wang , “Single‐Shot Echo Planar Time‐Resolved Imaging for Multi‐Echo Functional MRI and Distortion‐Free Diffusion Imaging,” Magnetic Resonance in Medicine 93, no. 3 (2025): 993–1013. 39428674 10.1002/mrm.30327 PMC11680730

[41] Z. Hu , A. J. L. Berman , Z. Dong , et al., “Reduced Physiology‐Induced Temporal Instability Achieved With Variable‐Flip‐Angle Fast Low‐Angle Excitation Echo‐Planar Technique With Multishot Echo Planar Time‐Resolved Imaging,” Magnetic Resonance in Medicine 93, no. 2 (2025): 597–614. 39323238 10.1002/mrm.30301 PMC11661687

[42] F. Wang , Z. Dong , L. L. Wald , J. R. Polimeni , and K. Setsompop , “Simultaneous Pure T2 and Varying T2'‐Weighted BOLD fMRI Using Echo Planar Time‐Resolved Imaging for Mapping Cortical‐Depth Dependent Responses,” NeuroImage 245 (2021): 118641. 34655771 10.1016/j.neuroimage.2021.118641 PMC8820652

[43] Z. P. Liang , “Spatiotemporal Imaging With Partially Separable Functions,” Joint Meeting of the 6th International Symposium on Noninvasive Functional Source Imaging of the Brain and Heart and the International Conference on Functional Biomedical Imaging, 2007.

[44] J. I. Tamir , M. Uecker , W. Chen , et al., “T(2) Shuffling: Sharp, Multicontrast, Volumetric Fast Spin‐Echo Imaging,” Magnetic Resonance in Medicine 77, no. 1 (2017): 180–195. 26786745 10.1002/mrm.26102 PMC4990508

[45] J. D. Trzasko and A. Manduca , “Local Versus Global Low‐Rank Promotion in Dynamic MRI Series Reconstruction,” 2010.

[46] P. J. Shin , P. E. Larson , M. A. Ohliger , et al., “Calibrationless Parallel Imaging Reconstruction Based on Structured Low‐Rank Matrix Completion,” Magnetic Resonance in Medicine 72, no. 4 (2014): 959–970. 24248734 10.1002/mrm.24997 PMC4025999

[47] J. Berglund and M. Skorpil , “Multi‐Scale Graph‐Cut Algorithm for Efficient Water‐Fat Separation,” Magnetic Resonance in Medicine 78, no. 3 (2017): 941–949. 27714826 10.1002/mrm.26479

[48] C. Y. Liu , C. A. McKenzie , H. Yu , J. H. Brittain , and S. B. Reeder , “Fat Quantification With IDEAL Gradient Echo Imaging: Correction of Bias From T(1) and Noise,” Magnetic Resonance in Medicine 58, no. 2 (2007): 354–364. 17654578 10.1002/mrm.21301

[49] W. Lu , H. Yu , A. Shimakawa , M. Alley , S. B. Reeder , and B. A. Hargreaves , “Water‐Fat Separation With Bipolar Multiecho Sequences,” Magnetic Resonance in Medicine 60, no. 1 (2008): 198–209. 18581362 10.1002/mrm.21583

[50] E. K. Brodsky , J. H. Holmes , H. Yu , and S. B. Reeder , “Generalized k‐Space Decomposition With Chemical Shift Correction for Non‐Cartesian Water‐Fat Imaging,” Magnetic Resonance in Medicine 59, no. 5 (2008): 1151–1164. 18429018 10.1002/mrm.21580

[51] D. Hernando , P. Kellman , J. Haldar , and Z. P. Liang , “Robust Water/Fat Separation in the Presence of Large Field Inhomogeneities Using a Graph Cut Algorithm,” Magnetic Resonance in Medicine 63, no. 1 (2010): 79–90. 19859956 10.1002/mrm.22177 PMC3414226

[52] H. Yu , A. Shimakawa , C. A. McKenzie , E. Brodsky , J. H. Brittain , and S. B. Reeder , “Multiecho Water‐Fat Separation and Simultaneous R2* Estimation With Multifrequency Fat Spectrum Modeling,” Magnetic Resonance in Medicine 60, no. 5 (2008): 1122–1134. 18956464 10.1002/mrm.21737 PMC3070175

[53] M. A. Griswold , P. M. Jakob , R. M. Heidemann , et al., “Generalized Autocalibrating Partially Parallel Acquisitions (GRAPPA),” Magnetic Resonance in Medicine 47, no. 6 (2002): 1202–1210. 12111967 10.1002/mrm.10171

[54] S. Boyd , “Distributed Optimization and Statistical Learning via the Alternating Direction Method of Multipliers,” Foundations and Trends in Machine Learning 3, no. 1 (2010): 1–122.

[55] Z. Tan , C. Unterberg‐Buchwald , M. Blumenthal , et al., “Free‐Breathing Liver Fat, R(2)* and B(0) Field Mapping Using Multi‐Echo Radial FLASH and Regularized Model‐Based Reconstruction,” IEEE Transactions on Medical Imaging 42, no. 5 (2023): 1374–1387. 37015368 10.1109/TMI.2022.3228075 PMC10368089

[56] X. Wang , Z. Tan , N. Scholand , V. Roeloffs , and M. Uecker , “Physics‐Based Reconstruction Methods for Magnetic Resonance Imaging,” Philosophical Transactions: Mathematical, Physical and Engineering Sciences 379, no. 2200 (2021): 20200196. 10.1098/rsta.2020.0196 PMC8107652 33966457

[57] X. Zhou , B. L. Daniel , B. A. Hargreaves , and P. K. Lee , “Distortion‐Free Water‐Fat Separated Diffusion‐Weighted Imaging Using Spatiotemporal Joint Reconstruction,” Magnetic Resonance in Medicine 92, no. 6 (2024): 2343–2357. 39051729 10.1002/mrm.30221 PMC12383488

[58] Z. Dong , F. Wang , T. G. Reese , et al., “Tilted‐CAIPI for Highly Accelerated Distortion‐Free EPI With Point Spread Function (PSF) Encoding,” Magnetic Resonance in Medicine 81, no. 1 (2019): 377–392. 30229562 10.1002/mrm.27413 PMC6258292

[59] A. R. Pineda , S. B. Reeder , Z. Wen , and N. J. Pelc , “Cramer‐Rao Bounds for Three‐Point Decomposition of Water and Fat,” Magnetic Resonance in Medicine 54, no. 3 (2005): 625–635. 16092102 10.1002/mrm.20623

[60] H. Kim , S. E. Taksali , S. Dufour , et al., “Comparative MR Study of Hepatic Fat Quantification Using Single‐Voxel Proton Spectroscopy, Two‐Point Dixon and Three‐Point IDEAL,” Magnetic Resonance in Medicine 59, no. 3 (2008): 521–527. 18306404 10.1002/mrm.21561 PMC2818363

[61] D. J. Larkman , J. V. Hajnal , A. H. Herlihy , G. A. Coutts , I. R. Young , and G. Ehnholm , “Use of Multicoil Arrays for Separation of Signal From Multiple Slices Simultaneously Excited,” Journal of Magnetic Resonance Imaging: JMRI 13, no. 2 (2001): 313–317. 11169840 10.1002/1522-2586(200102)13:2<313::aid-jmri1045>3.0.co;2-w

[62] K. Setsompop , B. A. Gagoski , J. R. Polimeni , T. Witzel , V. J. Wedeen , and L. L. Wald , “Blipped‐Controlled Aliasing in Parallel Imaging for Simultaneous Multislice Echo Planar Imaging With Reduced g‐Factor Penalty,” Magnetic Resonance in Medicine 67, no. 5 (2012): 1210–1224. 21858868 10.1002/mrm.23097 PMC3323676

[63] Z. Dong , F. Wang , X. Ma , E. Dai , Z. Zhang , and H. Guo , “Motion‐Corrected k‐Space Reconstruction for Interleaved EPI Diffusion Imaging,” Magnetic Resonance in Medicine 79, no. 4 (2018): 1992–2002. 28771867 10.1002/mrm.26861

[64] L. Feng , L. Axel , H. Chandarana , K. T. Block , D. K. Sodickson , and R. Otazo , “XD‐GRASP: Golden‐Angle Radial MRI With Reconstruction of Extra Motion‐State Dimensions Using Compressed Sensing,” Magnetic Resonance in Medicine: Official Journal of the Society of Magnetic Resonance in Medicine/Society of Magnetic Resonance in Medicine 75, no. 2 (2016): 775–788. 10.1002/mrm.25665 PMC4583338 25809847

