# Supplementary material for: Water/Fat Separated Echo Planar Time‐Resolved Imaging (WFS‐EPTI) for Distortion‐Free Multi‐Contrast MRI
Source: Magn Reson Med. 2026 Apr 9;96(2):580–95. doi: 10.1002/mrm.70355 (PMC13269247; doi:10.1002/mrm.70355)
Supplement: Supplementary file 1 — Figure S1: Signal evolution and subspace basis for a single‐peak fat spectrum (a) and a multi‐peak fat spectrum (b) for odd and even echoes. The multi‐peak model includes fat components with chemical shifts at 3.80, 3.40, 2.60, 1.94, 0.39, −0.60 ppm, while the single‐peak model only considers the main peak at 3.40 ppm. Note that, during subspace basis generation, the top panel uses fixed relative amplitudes of 0.087, 0.693, 0.128, 0.004, 0.039, 0.04 for the multi‐peak fat components, whereas the bottom panel shows the results when no constraints were imposed on the relative amplitudes of the fat components. The echo time and echo‐spacing were chosen such that in‐phase/out‐of‐phase condition can be achieved for the main fat peak. The signal evolutions were simulated with 44 echoes ranging from 12 to 63.6 ms, with an echo spacing of 1.2 ms. The left column presents simulated real and imaginary of water (red) and fat (blue) signals across echo times. The middle column illustrates the first three temporal basis components derived from singular value decomposition (SVD). The right column shows the corresponding singular value spectra (coefficients), with the number of significant basis components indicated by the red dashed lines. Figure S2: WFS‐EPTI reconstructions simulated with a numerical water/fat phantom. Data were simulated using a single‐peak fat spectrum (a) and a multi‐peak fat spectrum (b). Specifically, the data simulated with the 6‐peak fat model assumed relative amplitudes of 0.087, 0.693, 0.128, 0.004, 0.039, and 0.04, respectively. The phantom's proton density, proton density fat fraction (PDFF), and T2* values were used to generate fully sampled WFS‐EPTI data, along with simulated B0 maps. The data were simulated with 44 echoes ranging from 12 to 63.6 ms, with an echo spacing of 1.2 ms. The data were reconstructed using either simulated basis assuming perfect in‐phase/out‐of‐phase conditions (i.e., no phase modulation due to chemical‐shift effects, with [file MRM-96-580-s001.docx]

Water/Fat Separated Echo Planar Time-resolved Imaging (WFS-EPTI) for distortion-free multi-contrast MRI

Zhangxuan Hu, Timothy G. Reese, Lawrence L. Wald, Jonathan R. Polimeni, Zijing Dong, Fuyixue Wang

***Supplementary Materials***

## Effects of multi-peak fat spectrum on the subspace reconstruction of WFS-EPTI

Fig. S1 shows the signal evolution and subspace basis for a single-peak fat spectrum (Fig. S1a) and a multi-peak fat spectrum (Fig. S1b) for odd and even echoes. The multi-peak spectrum (b) includes fat components with chemical shifts at 3.80, 3.40, 2.60, 1.94, 0.39, -0.60 ppm, while the single-peak spectrum only considers the main peak at 3.40 ppm. Note that, during subspace basis generation, the top panel uses fixed relative amplitudes of 0.087, 0.693, 0.128, 0.004, 0.039, 0.04 for the multi-peak fat components, whereas the bottom panel shows the results when no constraints were imposed on the relative amplitudes of the fat components, which we view as a more general and practical formulation, since the relative amplitudes may vary in vivo due to differences in fat composition. For the single-peak spectrum, the in-phase/out-of-phase condition can be well achieved by choosing appropriate echo time and echo spacing based on the main fat peak, as reflected by the consistent T2* decay of fat and water signals in odd echoes and even echoes. Specifically, fat and water signals share the same phase (in-phase) for odd echoes but have opposite phases (out-of-phase) for even echoes. In this case, two subspace bases can accurately represent the signal evolutions (Fig. S1a). In contrast, for the multi-peak model, using echo time and echo spacing optimized only for the main peak cannot perfectly satisfy the in-phase/out-of-phase conditions for all spectral components, leading to additional phase evolution of the fat signal across echoes. Consequently, more bases are required, and they become more complex, especially when the relative amplitudes of the multiple spectral peaks of fat are not fixed during subspace basis generation. The data-driven basis proposed in this study (Figure 3) is therefore designed to capture these complex components while reducing the number of required bases by tailoring the basis generation to the characteristics of the acquired dataset (rather than relying on bases generated from the predefined multi-peak fat spectrum simulation as illustrated here).

Fig. S2 shows WFS-EPTI reconstructions simulated with a numerical water/fat phantom (2). Specifically, the data simulated with the 6-peak fat model assumed relative amplitudes of 0.087, 0.693, 0.128, 0.004, 0.039, and 0.04, respectively. For data simulated using a single-peak fat spectrum, WFS-EPTI with either simulated bases assuming perfect in-phase/out-of-phase conditions (i.e., no phase modulation due to chemical-shift effects for odd and even echoes, respectively) or data-driven basis (as obtained in auto-calibrated WFS-EPTI) can both reconstruct the images well and achieve good water/fat separation (Fig. S2a). For the data simulated with a multi-peak fat spectrum (Fig. S2b), reconstruction using simulated bases assuming perfect in-phase/out-of-phase conditions exhibit noticeable bias, mainly in regions containing fat, because the more complex fat signals can no longer be adequately represented by the bases. The data-driven bases perform better in this case, showing its capability to model part of the multi-spectrum fat signal evolution. Fig. S2c compares the nRMSE curves under different conditions.


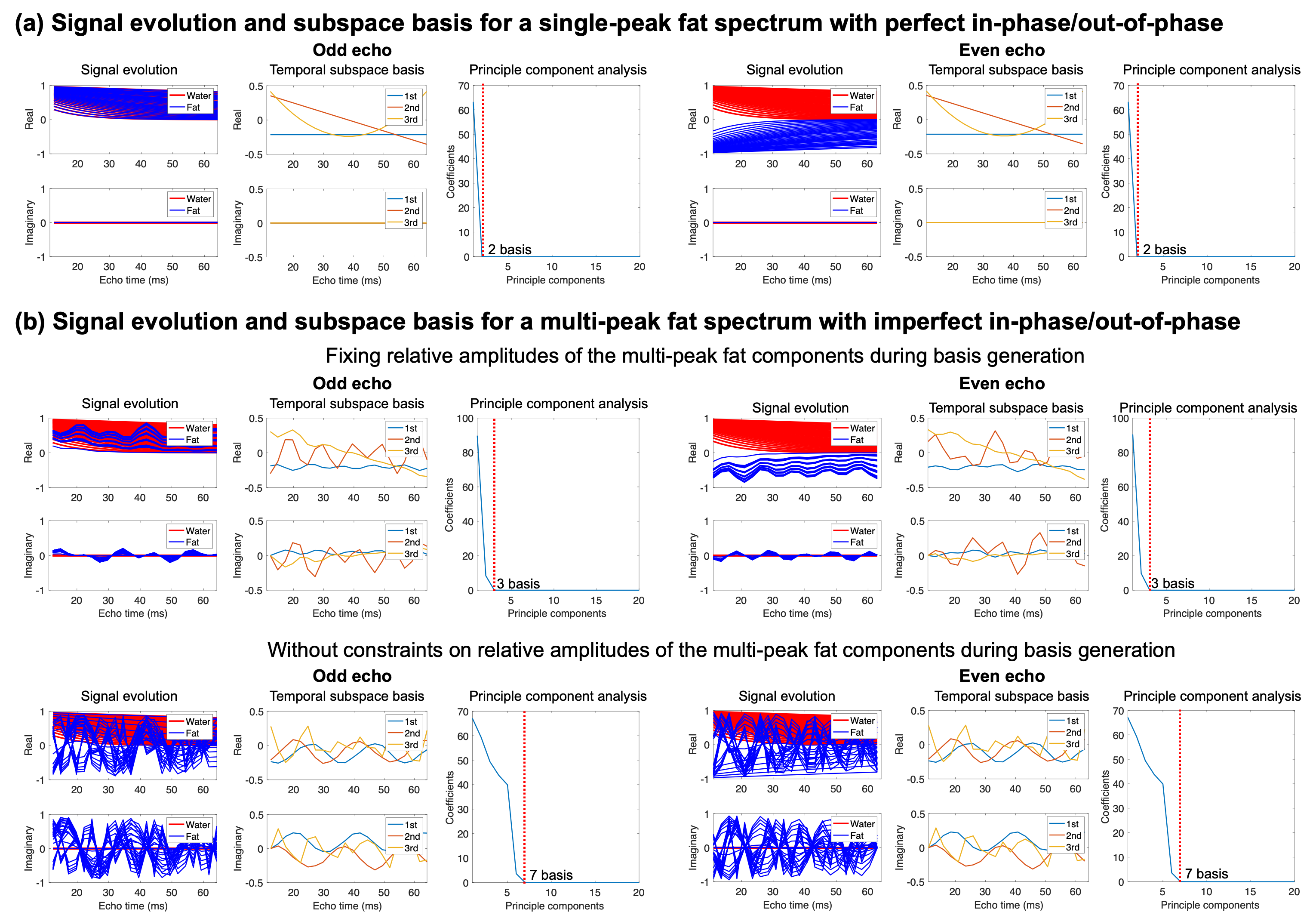


**Supplementary Figure S1:** Signal evolution and subspace basis for a single-peak fat spectrum (a) and a multi-peak fat spectrum (b) for odd and even echoes. The multi-peak model includes fat components with chemical shifts at 3.80, 3.40, 2.60, 1.94, 0.39, -0.60 ppm, while the single-peak model only considers the main peak at 3.40 ppm. Note that, during subspace basis generation, the top panel uses fixed relative amplitudes of 0.087, 0.693, 0.128, 0.004, 0.039, 0.04 for the multi-peak fat components, whereas the bottom panel shows the results when no constraints were imposed on the relative amplitudes of the fat components. The echo time and echo-spacing were chosen such that in-phase/out-of-phase condition can be achieved for the main fat peak. The signal evolutions were simulated with 44 echoes ranging from 12 ms to 63.6 ms, with an echo spacing of 1.2 ms. The left column presents simulated real and imaginary of water (red) and fat (blue) signals across echo times. The middle column illustrates the first three temporal basis components derived from singular value decomposition (SVD). The right column shows the corresponding singular value spectra (coefficients), with the number of significant basis components indicated by the red dashed lines.


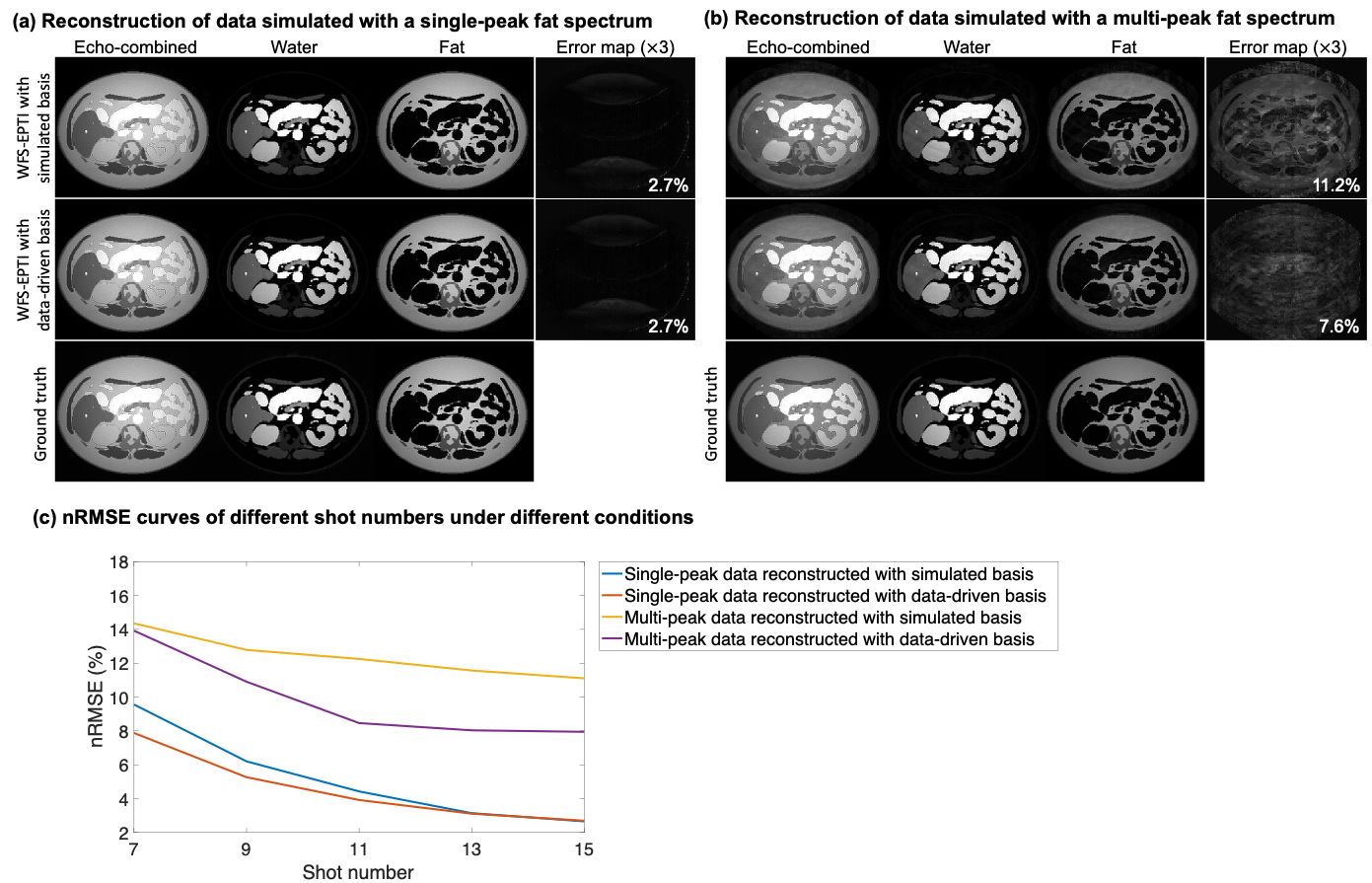


**Supplementary Figure S2:** WFS-EPTI reconstructions simulated with a numerical water/fat phantom. Data were simulated using a single-peak fat spectrum (a) and a multi-peak fat spectrum (b). Specifically, the data simulated with the 6-peak fat model assumed relative amplitudes of 0.087, 0.693, 0.128, 0.004, 0.039, and 0.04, respectively. The phantom’s proton density, proton density fat fraction (PDFF), and T2* values were used to generate fully sampled WFS-EPTI data, along with simulated B0 maps. The data were simulated with 44 echoes ranging from 12 ms to 63.6 ms, with an echo spacing of 1.2 ms. The data were reconstructed using either simulated basis assuming perfect in-phase/out-of-phase conditions (i.e., no phase modulation due to chemical-shift effects, with separate bases for odd and even echoes, respectively) or data-driven basis (as obtained in auto-calibrated WFS-EPTI). Echo-combined images, and water/fat-separated images are shown, together with error maps of the reconstructed images relative to the ground-truth images. Five acceleration factors were simulated, corresponding to acquisitions using 7, 9, 11, 13 and 15 shots. The results for the 15-shot case are shown in (a) and (b). The corresponding nRMSE curves under different conditions are shown in (c).

## Results of Experiment 3 in three additional subjects

**
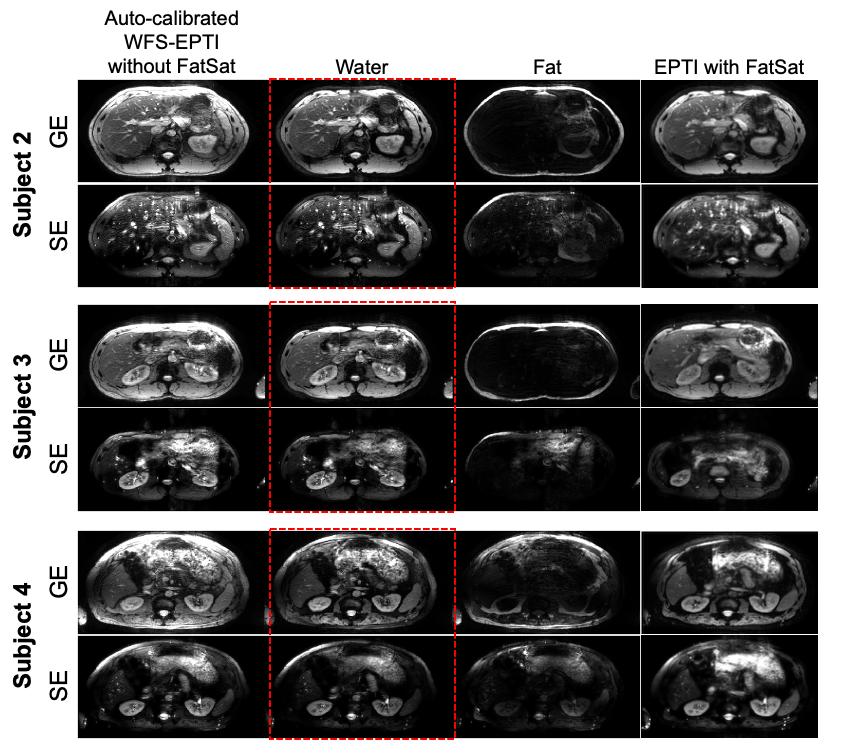
**

**Supplementary Figure S3:** In-vivo human abdominal imaging results obtained using the proposed auto-calibrated WFS-EPTI technique in 3 additional subjects. Data were acquired using the same acquisition matrix but different field-of-views tailored to individual body sizes (subject 2: 400 $\times$ 280 mm^2^; subject 3: 344 $\times$ 240 mm^2^; subject 4: 344 $\times$ 240 mm^2^). Echo-combined, water-only and fat-only images reconstructed from auto-calibrated WFS-EPTI without fat saturation are shown with both gradient and spin echo contrast. Auto-calibrated WFS-EPTI images acquired with fat saturation are also provided as the references. The proposed approach yields water-only images with minimal fat signal artifacts and image quality comparable to that of the data acquired with fat saturation. Note that images with different contrasts were acquired in separate breath-holds and therefore may not correspond to the same locations in each subject. Artifacts due to large vessels can be observed in Subject 3.

**
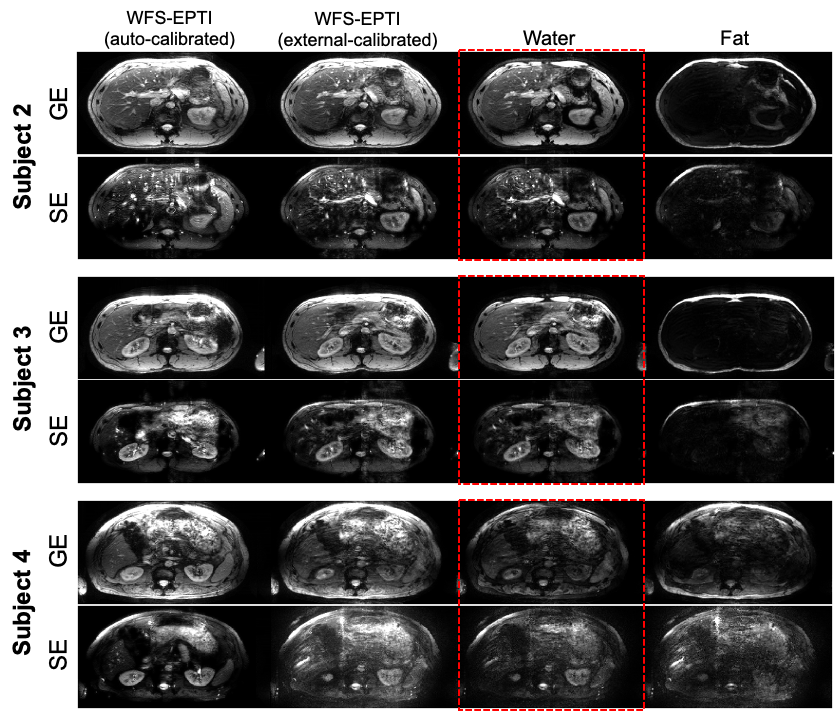
**

**Supplementary Figure S4:** In-vivo human abdominal imaging results obtained using auto-calibrated and external-calibrated WFS-EPTI in 3 additional subjects. Data were acquired using the same acquisition matrix but different field-of-views tailored to individual body sizes (subject 2: 400 $\times$ 280 mm^2^; subject 3: 344 $\times$ 240 mm^2^; subject 4: 344 $\times$ 240 mm^2^). Echo combined images, along with corresponding water-only and fat-only images, acquired using WFS-EPTI with external calibration, are presented. Corresponding images acquired using the auto-calibrated version of WFS-EPTI are also shown as the reference on the left. External-calibrated WFS-EPTI shows lower SNR compared with auto-calibrated ones due to less data samples, as expected. Note that images with different contrasts were acquired in separate breath-holds and therefore may not correspond to the same locations in each subject. WFS-EPTI with external calibration showed good performance in three out of the total four subjects, while one subject exhibited artifacts due to severe motion-induced mismatch between the calibration and image scans. Future work will address the vulnerability of external-calibration to motion-related misregistration of images anatomical structures across different breath-hold states.
